# Research on 3D crack segmentation of CT images of oil rock core

**DOI:** 10.1371/journal.pone.0258463

**Published:** 2021-10-14

**Authors:** Yongning Zou, Gongjie Yao, Jue Wang

**Affiliations:** 1 Key Laboratory of Optoelectronic Technology and System of the Education Ministry, Chongqing University, Chongqing, China; 2 Engineering Research Center of Industrial Computed Tomography Nondestructive Testing of the Education Ministry, Chongqing University, Chongqing, China; Bielefeld University, GERMANY

## Abstract

In this paper, we propose a framework for CT image segmentation of oil rock core. According to the characteristics of CT image of oil rock core, the existing level set segmentation algorithm is improved. Firstly, an algorithm of Chan-Vese (C-V) model is carried out to segment rock core from image background. Secondly the gray level of image background region is replaced by the average gray level of rock core, so that image background does not affect the binary segmentation. Next, median filtering processing is carried out. Finally, an algorithm of local binary fitting (LBF) model is executed to obtain the crack region. The proposed algorithm has been applied to oil rock core CT images with promising results.

## 1 Introduction

There are a large number of irregular and different scales of crack in the rock, which affect the macroscopic physical properties of the rock in varying degrees. Therefore, understanding and acquiring the crack structure characteristics of rocks is of great significance to the study of rocks. Industrial computed tomography (CT) has been widely applied for non-destructive testing (NDT) and non-destructive evaluation (NDE) [1]. Using CT scanning we can obtain images of rock cores. Obviously, CT images may be polluted by noise, which makes many problems in CT image segmentation, making some widely used methods unable to identify the target area [2]. The principal sources of noise in CT images arise during data acquisition. Therefore, accurate crack segmentation still remains one of the most challenging and studied problems in CT testing, due to the inevitable appearance of noises and artifacts.

Bhowmik et al. proposed a two-dimensional matched filtering technique followed by local entropy based thresholding, morphological operators and length filtering to detect and segment cracks from the cross-sectional images of rock [3]. Wang et al. adapted the mathematical morphology method based on the image threshold segmentation to obtain characteristic parameters of cracks of RSA [4]. The level set method, originally used as numerical technique for tracking interfaces and shapes, has been increasingly applied to image segmentation for over ten years [5]. Gao et al. proposed a novel statistical active contours method for using an arbitrary number of level set functions to segment the image into regions of the corresponding amount [6]. Jafarian et al. proposed an automatic model-based method using variational level set to segment the skull and fontanels from CT images [7]. Liu et al. extended the edge-based distance regularized level set evolution (DRLSE) model to a two-level-set formulation with the 0-level set and k-level set representing the endocardium and epicardium respectively [8]. Mandal et al. proposed a robust version of Chan and Vese algorithm [9] which is expected to achieve satisfactory segmentation performance, irrespective of the initial choice of the contour [10]. In the level set method, a contour is represented by the zero level set of a higher dimensional level set function. With the level set function, image segmentation problem can be solved in a principled way based on well-established mathematical theories, including calculus of variations and PDE [11]. Semantic segmentation is also a popular segmentation method. Zhu et al. [12] proposed a novel co-regularized unsupervised feature selection algorithm. Liu et al. [13] used the multithresholding image segmentation method to identify the cracks. In [14], the CT data was segmented into a binary volume using the automatic Otsu method.

C-V model is based on the assumption that the image is formed by two regions of approximately piecewise-constant intensities, and it can segment an object whose boundary is not necessary by gradient [15]. However, since C-V model only employs global intensity information of the image to detect boundary, it is difficult to segment cracks in the CT image of rock core, area of which is very small and the gray level of which is close to the gray level of the rock core. Owing to the fact that intensity inhomogeneity often occurs in real images, Li et al. [16,17] proposed Local Binary Fitting (LBF) energy model that draws upon intensity information in local regions at a controllable scale. The LBF model utilizes the local intensity means inside and outside of the contours to guide the evolution of the level set function. To deal with intensity inhomogeneity in image segmentation, Li et al. [18] proposed a variational level set framework for segmentation and bias correction of images with intensity inhomogeneity. Wang et al. [19] presented a new region-based active contour model in a variational level set formulation for image segmentation. They defined a local energy to characterize the fitting of the local Gaussian distribution. The local Gaussian distribution fitting (LGDF) energy is incorporated into a variational level set formulation with a level set regularization term. By using local region information, these methods can usually segment images with intensity inhomogeneity, although they are sometimes sensitive to the initial contour.

This paper studies the CT image of whole core with large diameter, so it is impossible to obtain a high-resolution image by using micro-CT. Therefore, we obtain low-resolution CT images. The cracks in CT images are very distinct to the human eye, but not so distinct for image segmentation algorithms. However, in CT images of oil rock cores, the cracks are not very distinct. If we directly use previous models, the segmentation results are often not satisfying. Based on the previous works [17–19], we propose a new 3D segmentation model for CT images of rock cores. First, we use C-V model to segment the entire rock cores involving cracks in the background. Secondly, the gray level of the background region is replaced with the average gay level of the rock core. Finally, LBF model is applied to the crack region segmentation. Comparative analysis of examples of different methods shows that our method is more accurate than the well-known level set models.

## 2 Related or Prior work

### 2.1 Chan-Vese (C-V) model

Chan and Vese [9] proposed an active contour model based on the piecewise constant case of Mumford–Shah functional [20]. Let Ω ⊂ ℝ^3^ be an open and bounded set, and let *I*: Ω → ℝ be a given image. Let Ω_1_, Ω_2_ be the distinct regions in Ω separated by a hyper-surface C, such that Ω = Ω_1_ ∪ C ∪ Ω_2_ and Ω_1_ ∩ Ω_2_ = ∅. C-V model is proposed to minimize the following energy functional:

$$F\left(C,c_{1},c_{2}\right)=\mu \cdot length(C)+\lambda_{1}\int_{\Omega_{1}}{\left|I(x)-c_{1}\right|}^{2}dx+\lambda_{2}\int_{\Omega_{2}}{\left|I(x)-c_{2}\right|}^{2}dx$$ (1)

where *λ*_1_, *λ*_2_ are fixed parameters and point ***x***(*x*_1_, *x*_2_, *x*_3_) ∈ Ω. The first term, with a weight *μ*, is introduced to regularize the surface *C*, while the last two terms are the data fitting terms. In the level set formulation, *C* is represented by the zero level set of a Lipschitz function *ϕ*: Ω → ℝ, and the 3D C-V model is formulated in terms of level set function as follows:

$$F\left(\phi ,c_{1},c_{2}\right)=\mu \int_{\Omega }|\nabla H\left({\left.\phi (x)\right|}^{2}dx+\lambda_{1}\int_{\Omega }{\left|I-c_{1}\right|}^{2}H\left(\phi (x)dx+\lambda_{2}\int_{\Omega_{2}}{\left|I-c_{2}\right|}^{2}(1-H(\phi (x))dx\right.\right.$$ (2)

where H is the Heaviside function. Image segmentation is therefore achieved by alternately iterating the level set function *ϕ* and the constants *c*_*i*_, *i* = 1, 2 that minimize the function *F*(*ϕ*, *c*_1_, *c*_2_). We can solve *c*_1_, *c*_2_ as follows:

$$c_{1}=\frac{\int H(\phi (\text{x}))\text{I}(\text{x})\text{dx}}{\int H(\phi (\text{x}))\text{dx}}$$ (3)

$$c_{2}=\frac{\int (1-\text{H}(\phi (\text{x})))\text{I}(\text{x})\text{dx}}{\int (1-\text{H}(\phi (\text{x})))\text{dx}}$$ (4)

By minimizing the energy functional (2) in terms of the level set function using the gradient descent method, we obtain the gradient descent flow

$$\left\{\begin{array}{l} \frac{\partial \phi }{\partial t}=\delta_{\varepsilon }(\phi )\left[\mu div\left(\frac{\nabla \phi }{|\nabla \phi |}\right)-v-{\left|I-c_{1}\right|}^{2}+{\left|I-c_{2}\right|}^{2}\right]\\ \phi (0,x)=\phi_{0}(x) \end{array}\right.$$ (5)

where δ(·) is the Dirac delta function.

### 2.2 Local Binary Fitting (LBF) model

In real images, intensity inhomogeneity often occurs. However, C-V model is based on the assumption of intensity homogeneity. In order to solve this kind of problem, Li et al. [16,17] proposed a local binary fitting model using intensity information in local regions. Consider a gray-level image *I*: Ω → ℝ. Let Ω_1_, Ω_2_ be the distinct regions in Ω. For a given point **x** ∈ Ω, a local fitting energy is defined as:

$$\varepsilon_{\text{x}}^{ft}\left(\phi ,{\text{c}}_{1}(\text{x}),{\text{c}}_{2}(\text{x})\right)=\sum_{i=1}^{2}\lambda_{i}\int_{\Omega_{i}}K_{\sigma }(\text{x}-\text{y}){\left|I(\text{y})-{\text{c}}_{i}(\text{x})\right|}^{2}d\text{y}$$ (6)

where *λ*_1_ and *λ*_1_ are positive constants, and *K*_*σ*_ is the Gaussian kernel function (*σ* denotes its standard deviation) with a localization property that *K*_*σ*_ (**x** − **y**) decreases and approaches to zero as **y** goes way from the center point **x**, *I*(**y**) denotes the image intensity of point **y**, and the fitting values c_1_(**x**) and c_2_(**x**) are two values that approximate image intensities in Ω_1_, Ω_2_. Combining the contour length energy term together, the total LBF energy functional in the image domain Ω is defined as follows:

$$\varepsilon^{LBF}\left(\phi ,{\text{c}}_{1},{\text{c}}_{2}\right)=\int \varepsilon_{X}^{ft}\left(\phi ,{\text{c}}_{1}(\text{x}),{\text{c}}_{2}(\text{x})\right)dx+v\int |\nabla H(\phi (\text{x}))|dx$$ (7)

To maintain stable evolution of the level set function, LBF model employs a level set regularization term

$$\text{P}(\phi )=\int \frac{1}{2}{\left(\left|\nabla \phi (\text{x})\right|-1\right)}^{2}dx$$ (8)

Therefore, the goal of the LBF model is to minimize the energy functional

$${\text{F}}^{\text{LBF}}\left(\phi ,{\text{c}}_{1},{\text{c}}_{2}\right)=\varepsilon^{LBF}\left(\phi ,{\text{c}}_{1},{\text{c}}_{2}\right)+\mu \text{P}(\phi )$$ (9)

where *μ* is a positive constant. By minimizing this energy functional in terms of c_1_, c_2_, we can solve c_1_, c_2_ as follows:

$$c_{1}(\text{x})=\frac{\int K_{\sigma }(\text{x}-\text{y})\text{H}(\phi (\text{y}))\text{I}(\text{y})\text{dy}}{\int K_{\sigma }(\text{x}-\text{y})\text{H}(\phi (\text{y}))\text{dy}}$$ (10)

$$c_{2}(\text{x})=\frac{\int K_{\sigma }(\text{x}-\text{y})(1-\text{H}(\phi (\text{y})))\text{I}(\text{y})\text{dy}}{\int K_{\sigma }(\text{x}-\text{y})(1-\text{H}(\phi (\text{y})))\text{dy}}$$ (11)

Minimizing the energy functional F^LBF^(*ϕ*, c_1_, c_2_) with respect to *ϕ* using the standard gradient descent method, we obtain the vibrational level set formulation as follows:

$$\frac{\partial \phi }{\partial t}=\delta (\phi )\left(-\lambda_{1}{\text{e}}_{1}+\lambda_{2}{\text{e}}_{2}+v\;\text{div}\left(\frac{\nabla \phi }{\left|\nabla \phi \right|}\right)\right)+\mu \left(\Delta \phi -\text{div}\left(\frac{\nabla \phi }{\left|\nabla \phi \right|}\right)\right)$$ (12)

where *e*_1_ and *e*_2_ are functions defined as follows:

$$e_{i}(\text{x})=\int K_{\sigma }(\text{y}-\text{x}){\left|I(\text{x})-{\text{c}}_{i}(\text{y})\right|}^{2}dy,\;i=1,2.$$ (13)

The fitting energy in (13) is region-scalable in the following sense. Large *σ* means large region size. Therefore, the C-V model can be considered as an extreme case of the LBF model for *σ* → ∞. The LBF model utilizes local image intensity may introduce many local minima in the energy functional. Consequently, the result of LBF model is more sensitive to initial contour than C-V model [21].

In real CT image of oil rock core, the gray levels of cracks are different from background that approximate zero. Due to the volume effect in CT system, their gray levels are bigger than zero. Thus, the C-V model cannot segment the cracks from rock core. The region-based model theoretically can segment the cracks, but in fact the background strongly affects the region-based evolution, result with lots of false regions that do not belong to cracks. In this paper we propose a synthesis method to obtain accurate segmentations of crack regions.

## 3 The proposed model

The CT image of oil core is composed of background area and core area. The grayscale difference between the two areas is large, so it is easy to separate them. Cracks are contained in the core area, with narrow shape and poor contrast. Direct use of these methods mentioned in the first section cannot accurately separate the crack region.

We first analyze the characteristics of the crack. The density of the crack is the same as that of the air, but the gray scale of the crack is different from that of the air on the CT image. Because of the point diffusion effect, the gray level of the crack is usually between the background gray level and the foreground gray level.

Secondly, we analyze the characteristics of level set method from formula (1) and formula (6). The essence of level set method is to divide the range of level set function into two. The first term of formula (1) is the regularization term, and the calculated value is related to the curvature of the level set function, that is, to the geometry of the target region. The second term and the third term are the intra-class variance of the target region and the background region respectively. If the first term is deleted from the formula, the result of segmentation is exactly the same as that of Ostu thresholding method. Using formula (1) on the core image can usually only obtain the sample area and air area. The LBF Algorithm expressed by formula (6) is similar to the local threshold segmentation algorithm. Although partial crack regions can be obtained, because of the binary segmentation of the level set method, the crack regions cannot be identified either with the background or with the object regions. If the background in the CT image is removed, there are only two kinds of gray areas: core solid area and core crack area. This satisfies the condition of level set binary segmentation.

Because of the above analysis, we proposed a new method to solve the crack segmentation of rock core CT data. Because the intensity of whole rock core does not change severely, it is easy for C-V model to segment the whole rock core. As a result of the influence of the background area, the LBF model cannot directly segment the cracks. However, the LBF model has obvious advantages to segment inhomogeneous gray-level images. The improved algorithm includes the merits of C-V model and LBF model. The steps of our algorithm are described as follows. Firstly, to use C-V model to segment the background region and the core region, secondly to replace the background gray level with the average gray level of the core region. This step can reduce the interference of the background region during LBF segmentation. At the end, LBF model is utilized to segment the rock core cracks. The first step contains two sub-steps: one sub-step is denoising on images by median filter. The other sub-step is to obtain initial segmentation via C-V model, so that we can locate the region of the core. The second step is a key step in the algorithm. We give the definition of the third step in formula (14).

$$\begin{array}{l} c_{1}=\frac{\int H(\phi (x))\text{I}(x)\text{d}x}{\int H(\phi (x))\text{d}x}\\ {\text{I}}_{\text{a}}=c_{1}\times (1-H(\phi (x))+\text{I}(x)\times H(\phi (x)) \end{array}$$ (14)

where *c*_1_ is the gray level of the rock core object and I_a_ is the image which background is adjusted.

**Fig 1:**
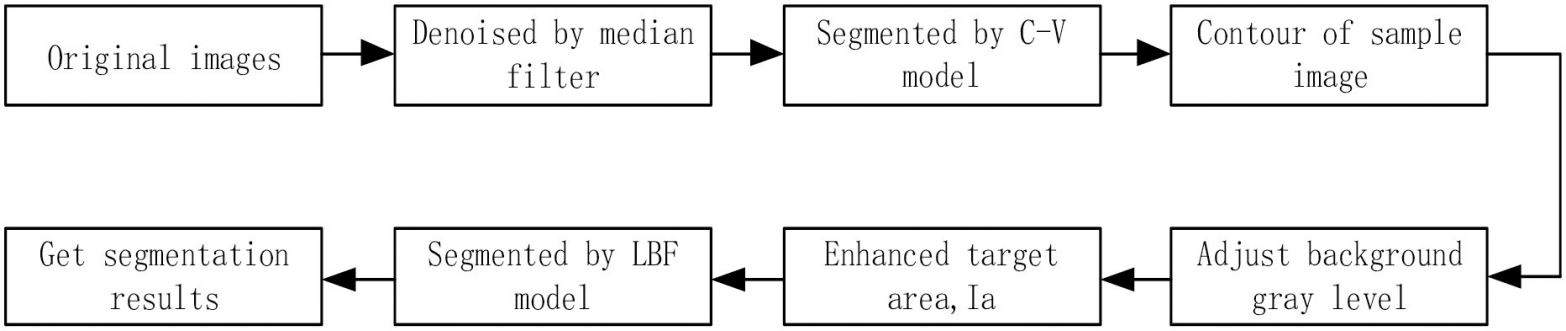
The flow chart of crack segment algorithm.

The flow chart of the improved rock core segmentation algorithm is shown in Fig 1.

The level set method has a good tolerance to noise, especially in the segmentation of large areas. However, due to the small size of the crack and the influence of noise, the denoising filter is conducive to the accurate segmentation of the crack. There are many denoising methods, this paper only uses a simple median filter, which is not necessarily the best in this application. In the future, we will study other filtering effects, such as Gaussian filtering, TV filtering and so on.

We make the pseudo-code of the proposed algorithm that is given as follows.

**Algorithm** 1: Level set segmentation of oil rock core CT image

**Input**: *I* ∈ ℝ^*3*^, parameters *λ*_*1*_, *λ*_*2*_, *μ*, *ν*, Δ*t*, *ε*, *σ* and number of iterations *N*_*iter*_.

**Output**: segmentation region *C*

1: Initialize level set function *ϕ*_*0*_.

2: **for** t = 1: *N*_*iter*_ do

3:  Calculate *c*_1_ using Eq (3).

4:  Calculate *c*_2_ using Eq (4).

5: Update *ϕ* using Eq (5).

6: **end** for

7: Obtain rock core region: *A* ← *binarization* (*ϕ*)

8: Number of pixels in core: *p* ← *sum*(*A*)

9: Sum of pixel intensity of core: *S* ← *sum*(*I* × *A*)

10: Average intensity of core: *V* ← *S*/*p*

11: Replace the background pixel intensity with *V*.

12: Reinitialize level set function *ϕ*_*0*_

13: **for** t = 1: *N*_*iter*_ do

14:  Calculate *c*_1_ using Eq (10).

15:  Calculate *c*_2_ using Eq (11).

16:  Calculate *ϕ* using Eq (8).

17:  Calculate fitting energy using Eq (13).

18:  Update *ϕ* using Eq (12).

19: **end** for

20: *C* ← *binarization* (*ϕ*)

21: Return segmentation result *C*.

## 4 Experimental results

**Fig 2:**
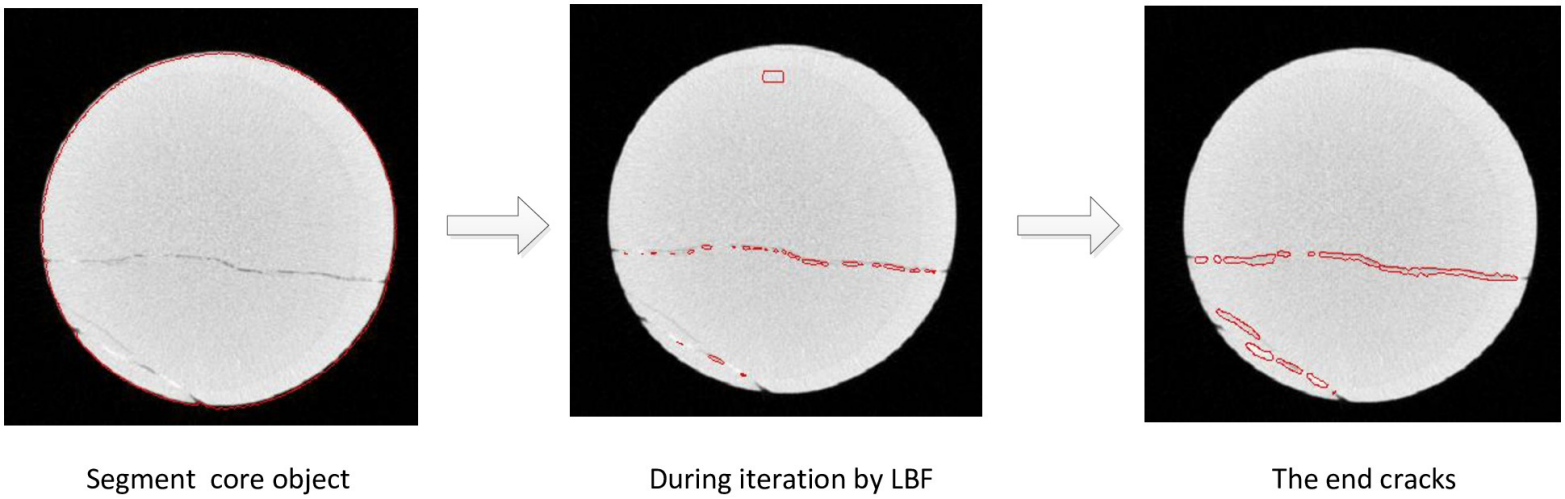
The end result of each phase of the algorithm.

**Fig 3:**
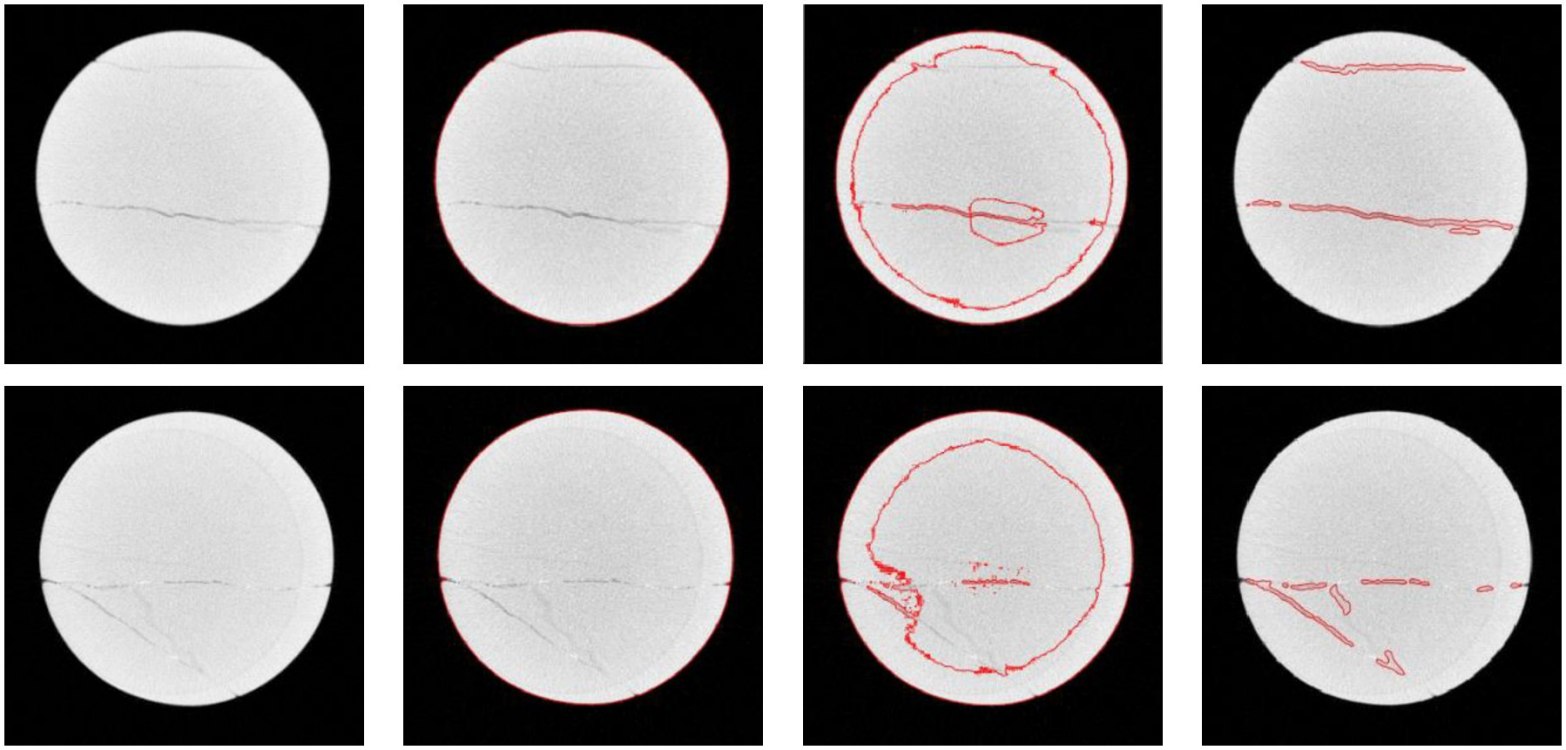
Segmentation results of the 2nd slice and the 48th slice of a core. From left to right, original images, C-V model. LBF model, the method proposed in this paper.

**Fig 4:**
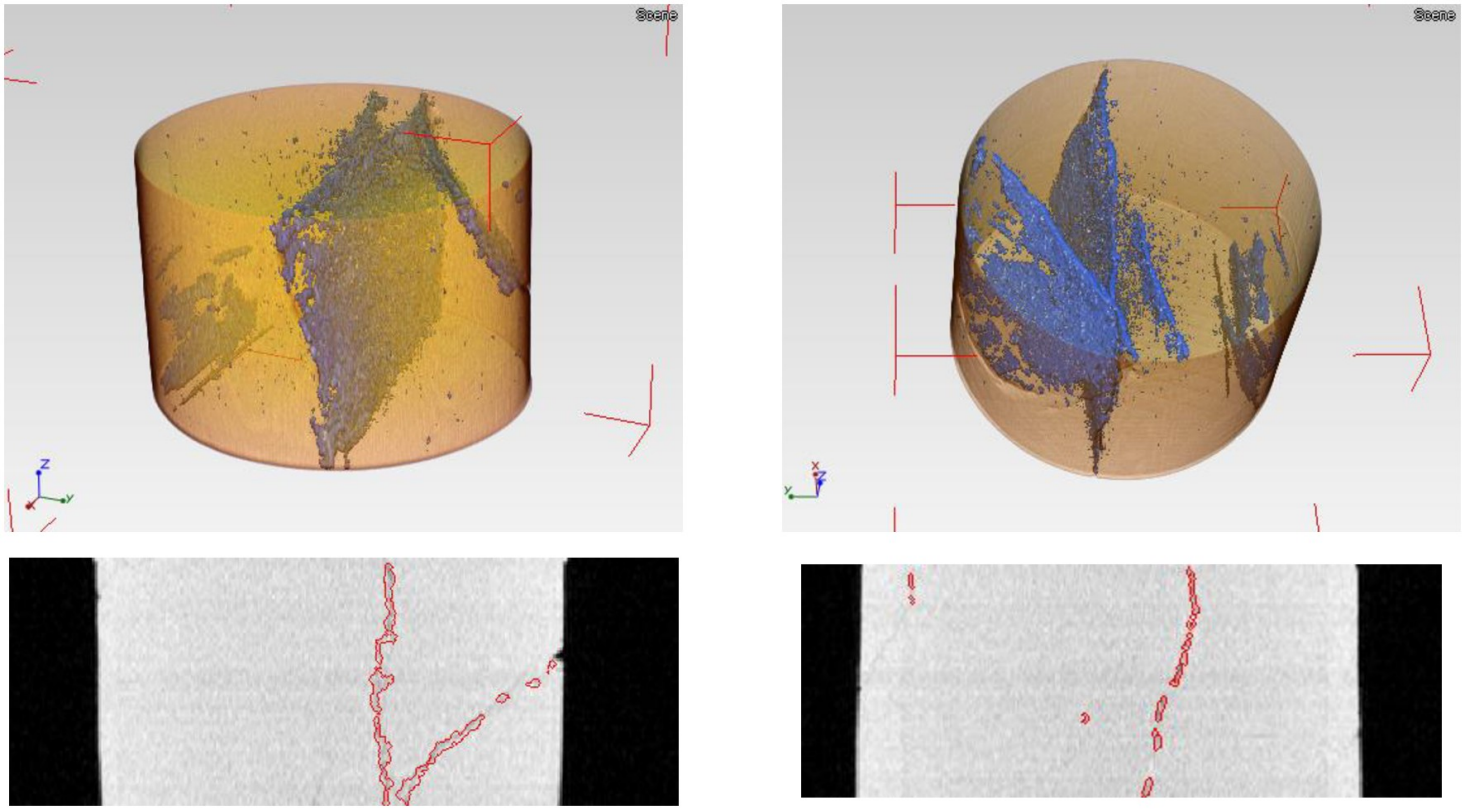
The volume visualization of the segmentation results of a core rock using our method.

We have tested our algorithm on three different test specimens. It has to be mentioned that the algorithm in this paper was implemented in Matlab on a PC with Windows 10, Intel (R)Core(TM) i5-4590 3.3GHz GPU, 8GB RAM and NVIDIA GeForce GTX 970 graphics card. Some of the test results are shown in Figs 3, 4, 6, 7 and 8. In Fig 2, we show the result of every processing step of our algorithm. The test results are compared with those of C-V model and LBF model. It should be emphasized that all parameters of C-V and LBF models are optimized empirically in the test.

### 4.1 Homogenous intensity oil rock core with narrow cracks

This section presents examples executed to assess the performance of the proposed method in comparison with C-V model and LBF model. We describe the data used for the experiment in this section. As we know, there is not unified framework for the validation of 3D oil rock core segmentation. We chose to consider oil rock core images with several cracks. So we can roughly judge if the method is effective. The first CT data we used is from a scanned rock core which intensity is roughly homogenous. This oil rock core was acquired from Tarim Oilfield. Its diameter is about 65mm. Its height is about 100mm. The X-ray energy of the CT employs 450kV. The focus spot size is about 1mm. The detector system is linear array structure with multi-pinhole collimator. The sampling time is 0.05s. The slice thickness is 1mm. The in-plane voxel size is 0.248mm×0.248mm. This CT data contains 59 CT slices. Each slice is 320 ×320 pixels. We compared our developed method with Local Binary Fitting (LBF) method and C-V model. Fig 3 includes segmentation results on two slices. The first row is the second slice of CT data, and the second row is the forty eighth slice of CT data. The first column is original images. The second column is segmentation results via C-V model. The third column is the segmentation results via LBF. The forth column is the segmentation results via the proposed method. In the experiment, μ is set to 0.01×255×255, time step Δ*t* is set to 0.1, λ_1_, λ_2_ is set to 1.0, *σ* is set to 6.0. These input parameters are empirical values obtained through a large number of tests. μ is generally chosen in interval [0.001x255x255, 0.01x255x255], and *σ* is generally chosen in [1, 10]. *N*_*iter*_ is chosen in [100, 200]. The sensitivity to the input parameters is one tenth of the selection range.

As shown in Fig 3, the fourth column is the segmentation result of the improved algorithm; crack segmentation effect is more accurate than C-V model and LBF model. Note that the C-V model can correctly segment the whole rock core region, but failed to segment any cracks that is exactly what we need. The LBF model also can segment the whole rock core region and a part of cracks, but including a large area of false cracks. The evolution of the upper image of the fourth column takes 25.0781 seconds. The evolution of the lower image of the fourth column takes up to 28.3249 seconds. To sum up, the improved level set crack detection algorithm can quickly and accurately detect the cracks in the oil core CT image, and can help the tester to further analyze the crack characteristics. The experiment verifies the effectiveness of the improved method proposed in this paper. Fig 4 shows the 3D visualization of the segmentation results using our improved method. In Fig 4, the first row is a 3D perspective rendering that doesn’t use a scale bar, because perspective projection causes the effect that near objects are big or far objects are small. If we want to measure the thickness of cracks in 3D scene, a 3D widget tool like a line segment is needed. The second row in Fig 4 is segmented slices along vertical planes of rock core. Inside the red contour is the crack area.

**Fig 5:**
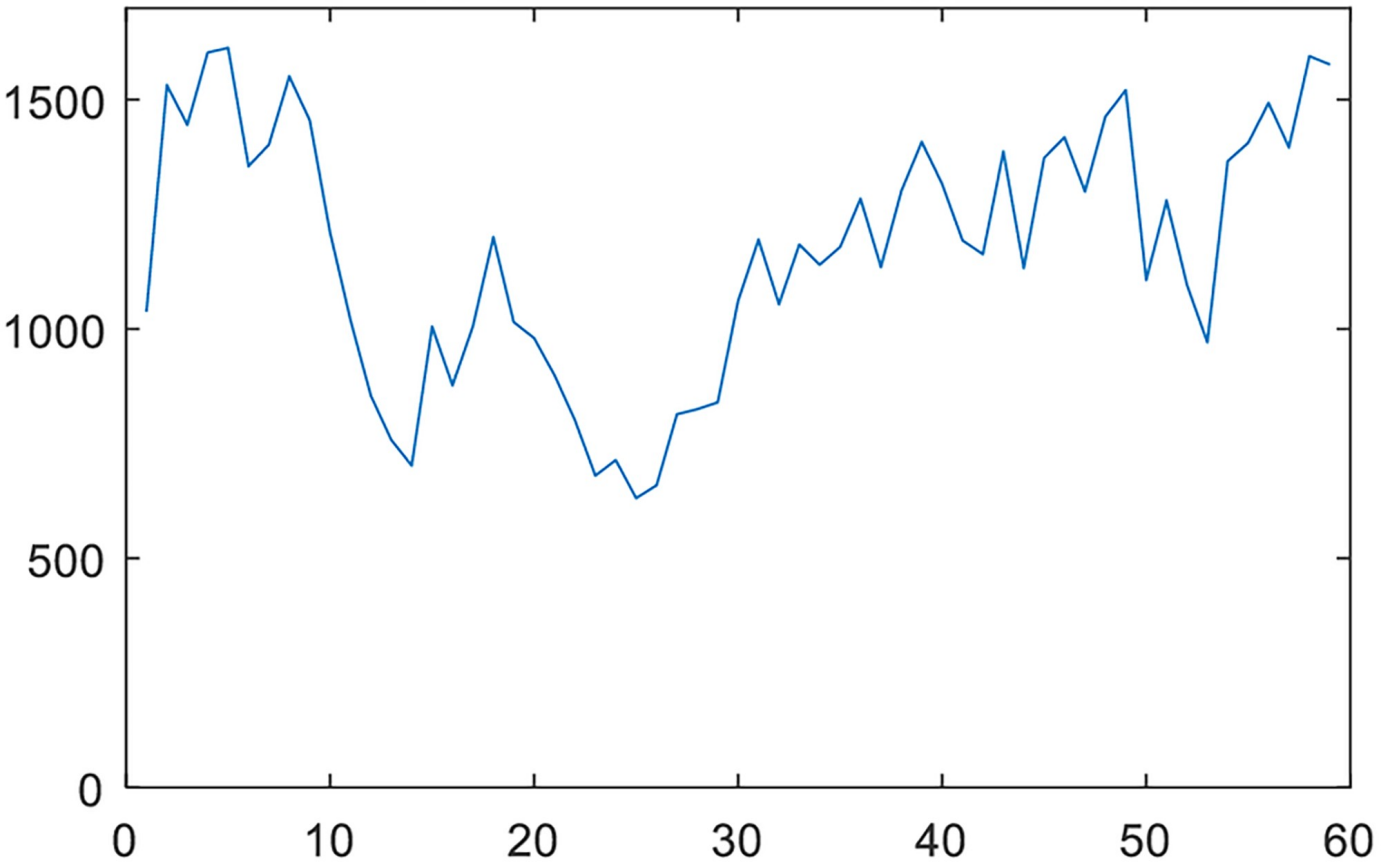
Change of the crack area of each slice.

In order to further verify the accuracy of crack segmentation, the crack area of each slice is calculated using the first rock sample. The Table 1 lists the minimum and maximum values of crack areas. The pixel physical width is 0.248mm. Fig 5 shows the change of crack area of each slice.

**Table 1:** Minimum and maximum values of crack areas.

	Minimum area	Maximum area
Crack area(pixel^2^)	631	1613
Crack area(mm^2^)	38.8	99.2

**Fig 6:**
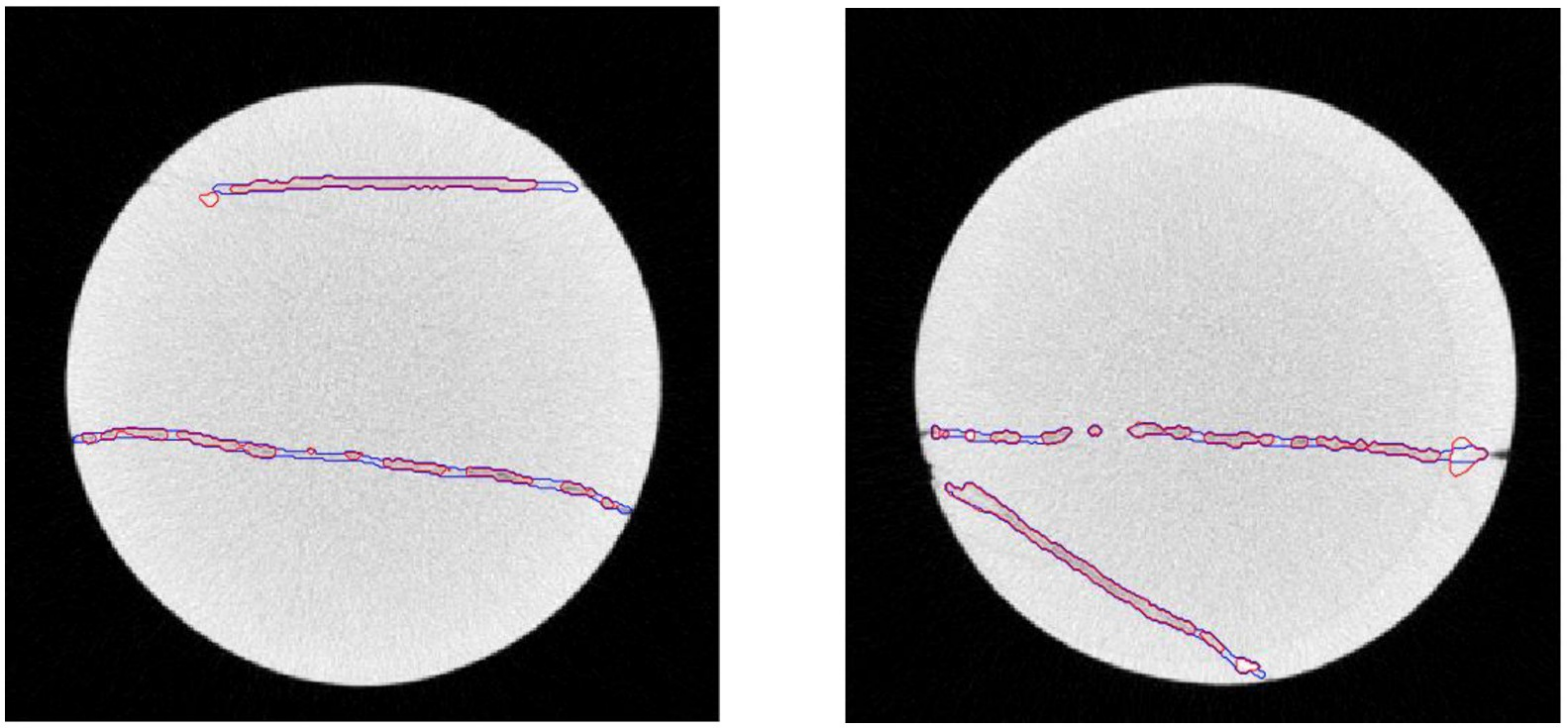
Display crack region boundary, the red is our method, the blue is ground truth.

The crack segmentation accuracy is analyzed by Intersection over Union (IoU) metric. In addition to using our method to segment the cracks, we also provide the ground truth crack area. Fig 6 shows the crack region boundary, the red is our method, the blue is ground truth. Table 2 shows the Segmentation result comparison with ground truth using IoU metric. The main reason that causes the discrepancy compared to ground truth is that the gray level of cracks is not consistent. The gray contrast is shallow where the crack is very narrow.

**Table 2:** Segmentation result comparison with ground truth using IoU metric.

Slice No.	Segmented	Ground truth	Intersection	Union	IoU
1	1038	1237	981	1294	75.8%
38	1347	1378	1239	1486	83.4%

### 4.2 Inhomogeneous intensity oil rock core with wide cracks

**Fig 7:**
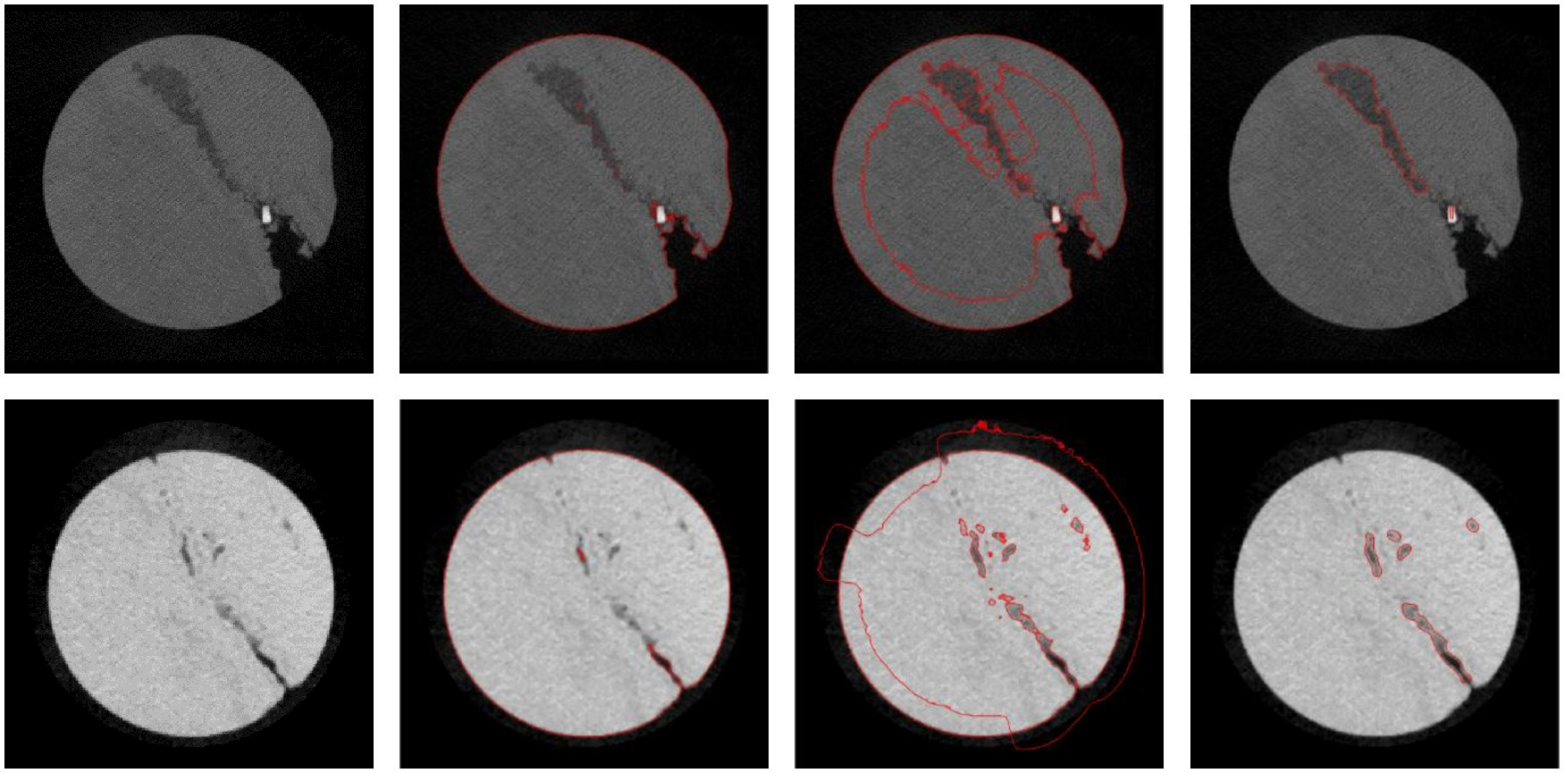
Segmentation results of the 2nd slice and the 48th slice of a core. From left to right, original images, C-V model, LBF model, the method proposed in this paper.

Due to being mixed with metal or other inhomogeneous material, some rock cores have inhomogeneous intensity. We made another experiment for this situation, which results are shown in Fig 7. The first row shows a rock core slice with a wide crack and metal inclusion. The second row shows a rock core slice with inhomogeneous intensity. The parameter σ is set to 4.0. The parameter *μ* is set to 0.001×255×255. In order to study the effect of our method, we compared the executing results of C-V model, LBF model, and the proposed algorithm. The last column in Fig 7 shows that the proposed algorithm can almost complete the crack segmentation. On the other side, the C-V and LBF model are not able to segment the cracks correctly. These results demonstrate the robustness of the proposed algorithm. The size of the bright area in the top row is small and does not affect the crack segmentation. If the bright area becomes larger, it may cause some problems. Statistics like C1, C2 play a role in LBP. Small area values do not interfere with statistics.

Like the last experiment, the C-V model successfully segmented the whole rock core, but not the cracks. Meanwhile, the LBF model segmented a large area of false cracks. Since the C-V model belongs to two-phase formulation, and the intensity of the cracks is different from the air intensity and the rock intensity, its segmentation result only contains two parts: air region and core region.

### 4.3 Segmentation on CT images of rock samples

**Fig 8:**
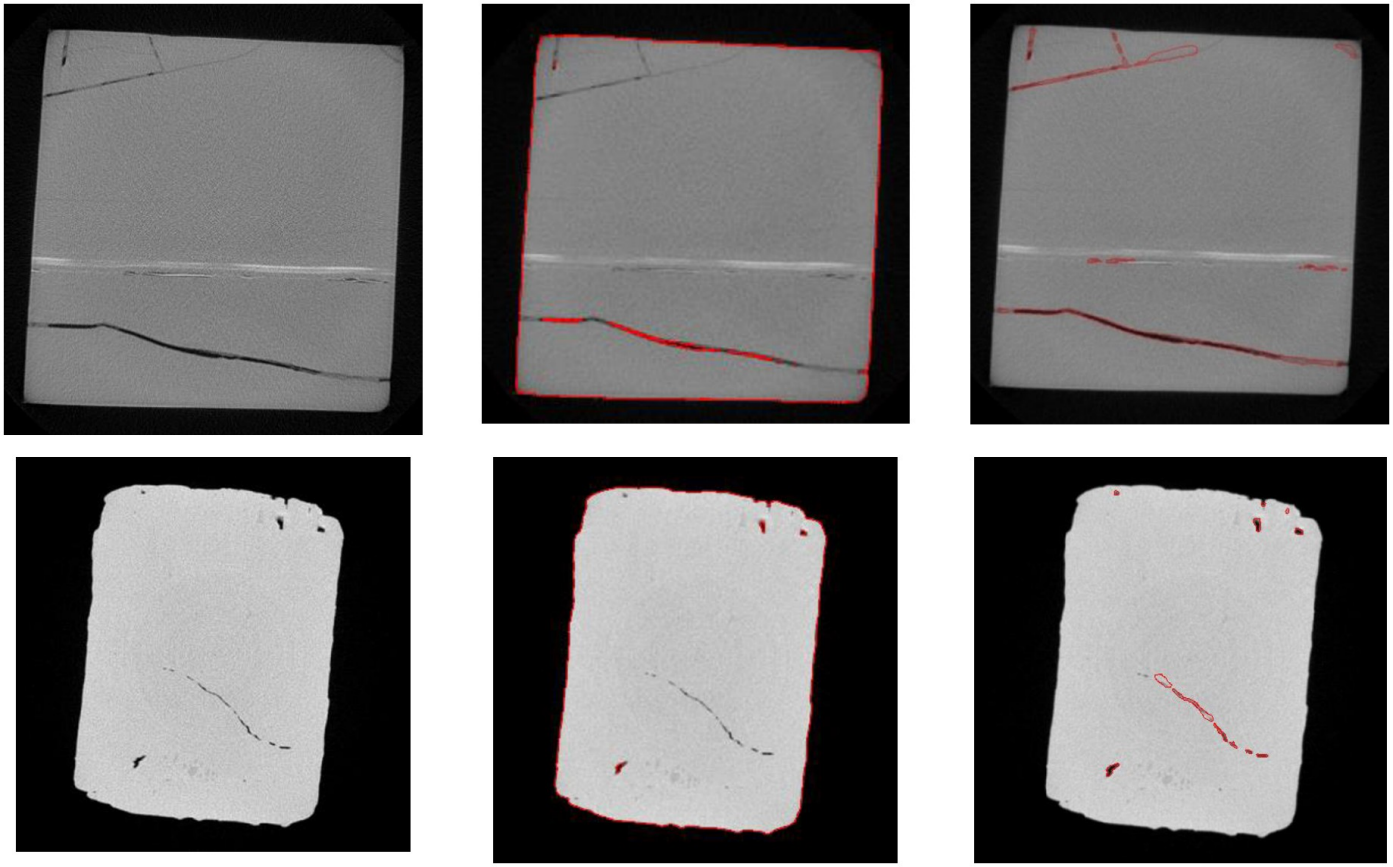
Segmentation results of rock samples. From left to right, original images, C-V model, the proposed method.

We collected CT images of other types of rock samples to test the effectiveness of the proposed method. Fig 8 shows the segmentation results of our algorithm and that of C-V model. The size of the first row is 528×528, and the size of the second row is 500×500. Due to the interference of gray level in the background region, CV model cannot extract finer cracks as shown in the second column. The proposed method can extract more complete cracks.

The proposed method also can be used to segment CT images of various samples and workpieces with cracks, air bubble, or inclusions. Though current proposed method has good effect on oil core segmentation, the challenge still exists. We will study the growth of cracks using semantic segmentation, and compare that result with other methods.

The data used in this paper is available at the following location on the URL: https://github.com/z213719/rock_core.git.

### 4.4 Challenges and future work

Our method has some shortcomings in practical application, which need to be overcome.

Level set methods need an initial level set function. In most cases, this initial level set function is arbitrary and will converge to the boundary of the object. If the convergence of different seed regions is different, we can give a rough seed region for a class of problems.

The proposed method requires that the core (once the background is removed) consist of two intensity populations, i.e. two classes of materials. If the core contains a variety of density materials, since the density of all materials is greater than the crack density, then we can regard all materials as an average material.

It is a challenge for Mumford-Shah functional that the narrow cracks don’t constitute a true single-material population. For severe inhomogeneous crack images, we need to reduce the inhomogeneity in the preprocessing stage.

Because of the iterative process, level set methods are computationally intensive, especially for 3D CT images. The running time of the first CT data is about 6 minutes. If the segmented CT volume data reaches gigabit voxels, the running time will be much larger. In order to create an efficient and fast segmentation technique, we plan to implement our algorithm on the high-performance computing device (GPU).

## 5 Conclusion

This paper presents an improved level set algorithm for 3D segmentation of rock core cracks. In order to overcoming the impact of image background, the algorithm utilizes different level set models. The key step is to adjust the original CT image background gray level with the rock core average gray level. This algorithm can be carried out automatically, so that it does not need a manual placement of markers or user interaction. It has been validated by various examples of segmentations of oil rock core CT images. Compared with C-V and LBF model, the improved method has the advantages of fast segmentation speed and much higher segmentation accuracy.

## Supporting information

S1 File (ZIP)
