# Supplementary figures and images for: Research on 3D crack segmentation of CT images of oil rock core (part 2 of 2)
Source: PLoS One. 2021 Oct 14;16(10):e0258463. doi: 10.1371/journal.pone.0258463 (PMC8516274; doi:10.1371/journal.pone.0258463)

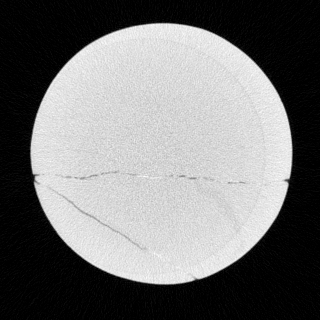

Supplement: S1 File — (ZIP) [file pone.0258463.s001.zip › oil rock cores/41.bmp]

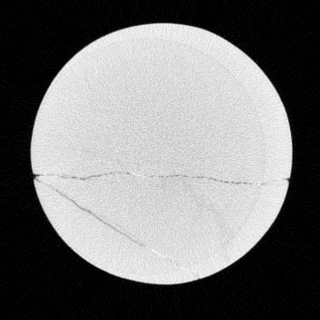

Supplement: S1 File — (ZIP) [file pone.0258463.s001.zip › oil rock cores/42.bmp]

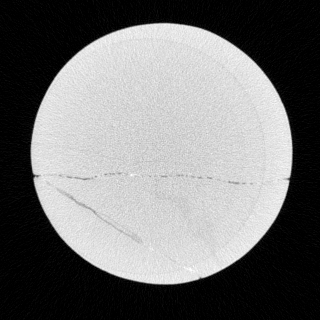

Supplement: S1 File — (ZIP) [file pone.0258463.s001.zip › oil rock cores/43.bmp]

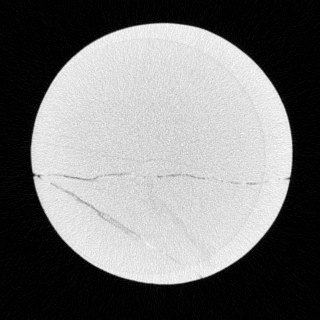

Supplement: S1 File — (ZIP) [file pone.0258463.s001.zip › oil rock cores/44.bmp]

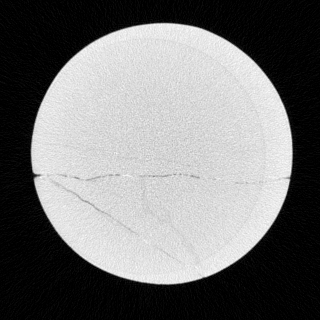

Supplement: S1 File — (ZIP) [file pone.0258463.s001.zip › oil rock cores/45.bmp]

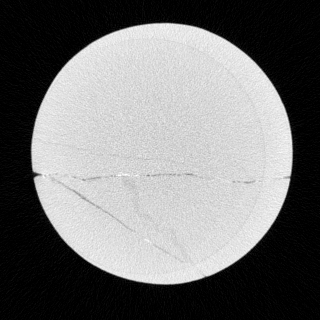

Supplement: S1 File — (ZIP) [file pone.0258463.s001.zip › oil rock cores/46.bmp]

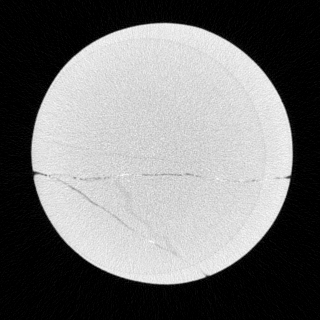

Supplement: S1 File — (ZIP) [file pone.0258463.s001.zip › oil rock cores/47.bmp]

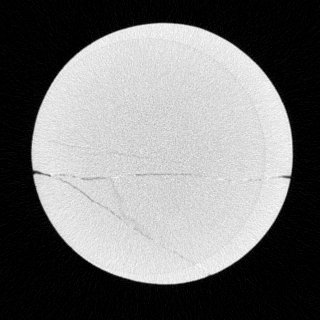

Supplement: S1 File — (ZIP) [file pone.0258463.s001.zip › oil rock cores/48.bmp]

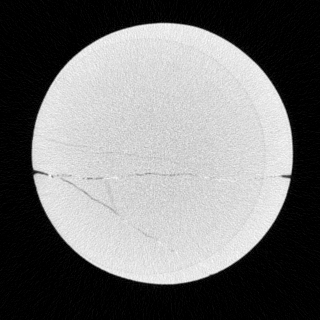

Supplement: S1 File — (ZIP) [file pone.0258463.s001.zip › oil rock cores/49.bmp]

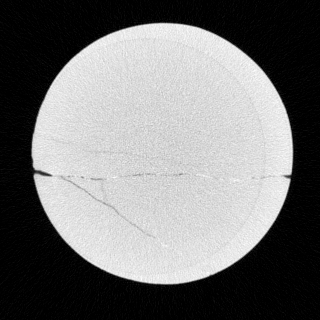

Supplement: S1 File — (ZIP) [file pone.0258463.s001.zip › oil rock cores/50.bmp]

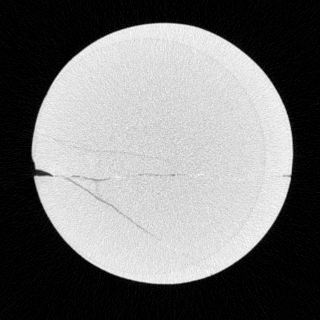

Supplement: S1 File — (ZIP) [file pone.0258463.s001.zip › oil rock cores/51.bmp]

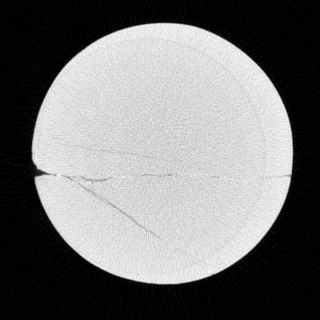

Supplement: S1 File — (ZIP) [file pone.0258463.s001.zip › oil rock cores/52.bmp]

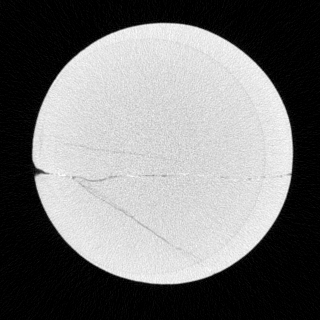

Supplement: S1 File — (ZIP) [file pone.0258463.s001.zip › oil rock cores/53.bmp]

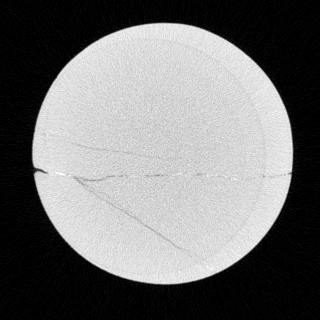

Supplement: S1 File — (ZIP) [file pone.0258463.s001.zip › oil rock cores/54.bmp]

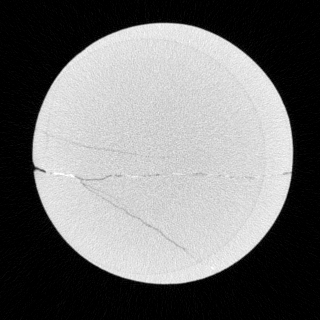

Supplement: S1 File — (ZIP) [file pone.0258463.s001.zip › oil rock cores/55.bmp]

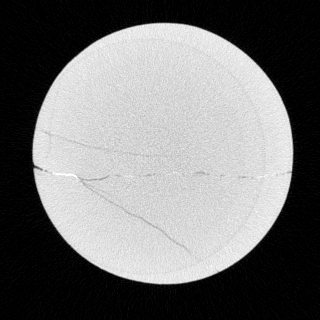

Supplement: S1 File — (ZIP) [file pone.0258463.s001.zip › oil rock cores/56.bmp]

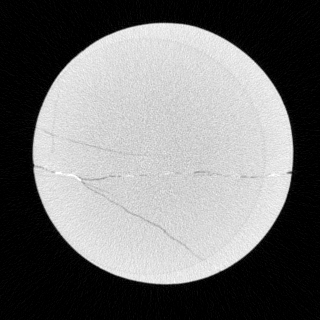

Supplement: S1 File — (ZIP) [file pone.0258463.s001.zip › oil rock cores/57.bmp]

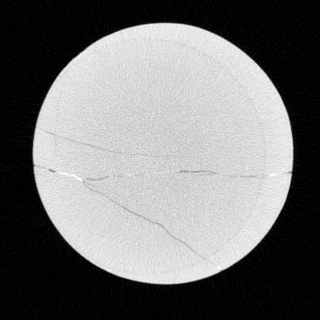

Supplement: S1 File — (ZIP) [file pone.0258463.s001.zip › oil rock cores/58.bmp]
