# Supplementary figures and images for: Research on 3D crack segmentation of CT images of oil rock core (part 1 of 2)
Source: PLoS One. 2021 Oct 14;16(10):e0258463. doi: 10.1371/journal.pone.0258463 (PMC8516274; doi:10.1371/journal.pone.0258463)

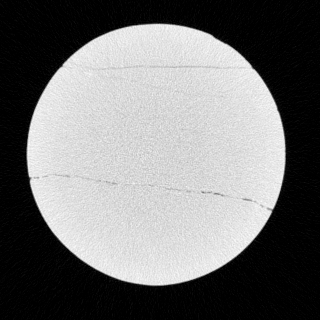

Supplement: S1 File — (ZIP) [file pone.0258463.s001.zip › oil rock cores/00.bmp]

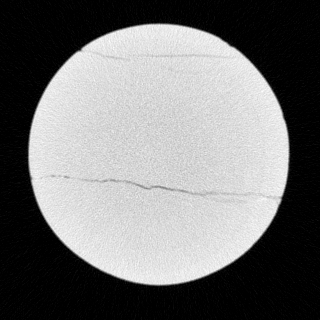

Supplement: S1 File — (ZIP) [file pone.0258463.s001.zip › oil rock cores/01.bmp]

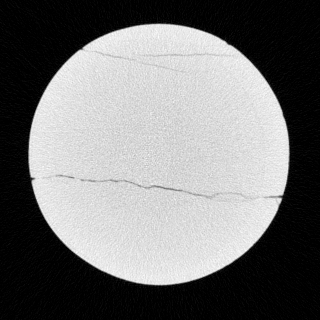

Supplement: S1 File — (ZIP) [file pone.0258463.s001.zip › oil rock cores/02.bmp]

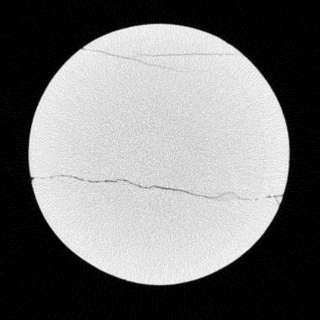

Supplement: S1 File — (ZIP) [file pone.0258463.s001.zip › oil rock cores/03.bmp]

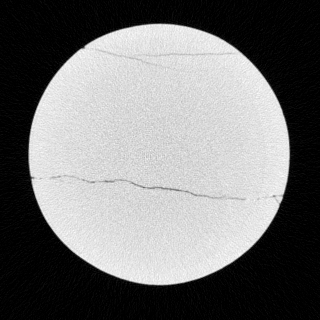

Supplement: S1 File — (ZIP) [file pone.0258463.s001.zip › oil rock cores/04.bmp]

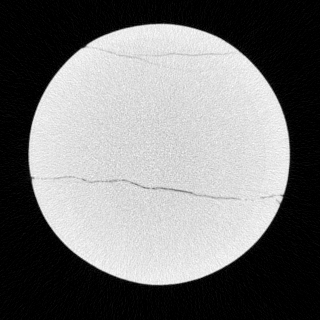

Supplement: S1 File — (ZIP) [file pone.0258463.s001.zip › oil rock cores/05.bmp]

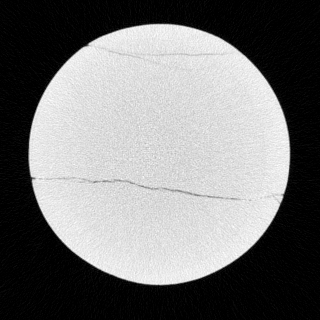

Supplement: S1 File — (ZIP) [file pone.0258463.s001.zip › oil rock cores/06.bmp]

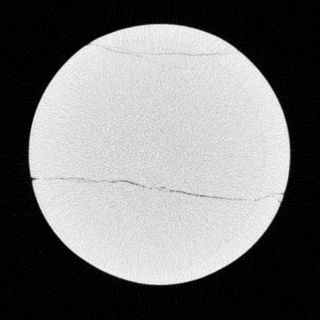

Supplement: S1 File — (ZIP) [file pone.0258463.s001.zip › oil rock cores/07.bmp]

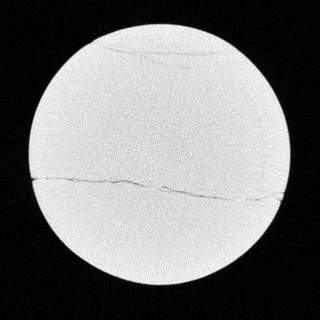

Supplement: S1 File — (ZIP) [file pone.0258463.s001.zip › oil rock cores/08.bmp]

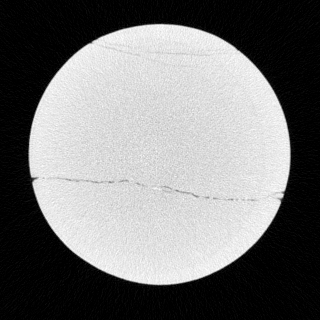

Supplement: S1 File — (ZIP) [file pone.0258463.s001.zip › oil rock cores/09.bmp]

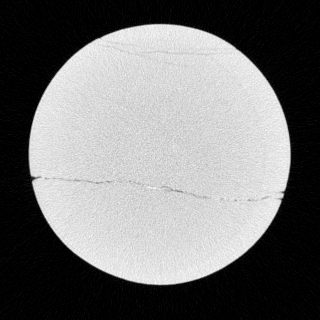

Supplement: S1 File — (ZIP) [file pone.0258463.s001.zip › oil rock cores/10.bmp]

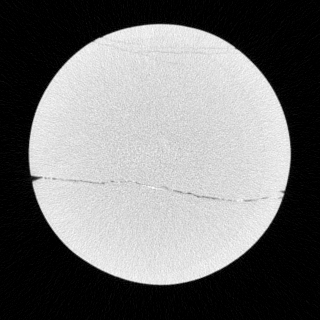

Supplement: S1 File — (ZIP) [file pone.0258463.s001.zip › oil rock cores/11.bmp]

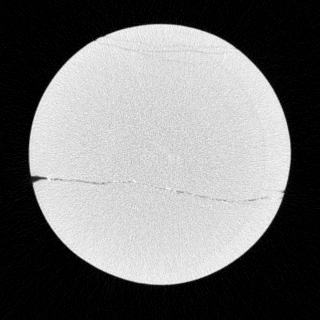

Supplement: S1 File — (ZIP) [file pone.0258463.s001.zip › oil rock cores/12.bmp]

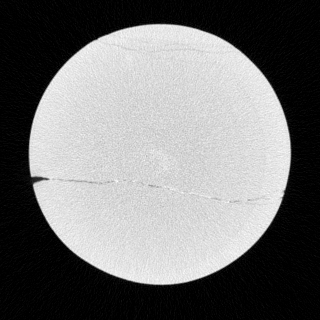

Supplement: S1 File — (ZIP) [file pone.0258463.s001.zip › oil rock cores/13.bmp]

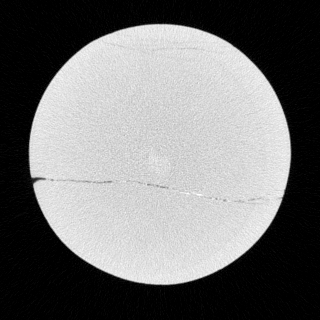

Supplement: S1 File — (ZIP) [file pone.0258463.s001.zip › oil rock cores/14.bmp]

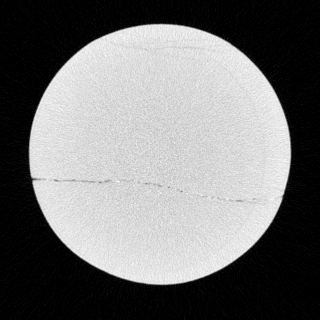

Supplement: S1 File — (ZIP) [file pone.0258463.s001.zip › oil rock cores/15.bmp]

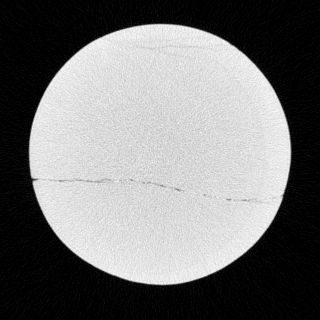

Supplement: S1 File — (ZIP) [file pone.0258463.s001.zip › oil rock cores/16.bmp]

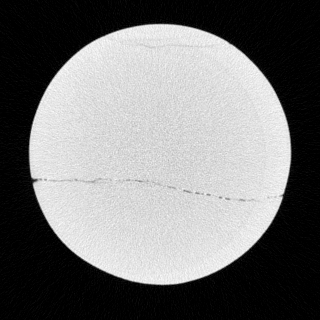

Supplement: S1 File — (ZIP) [file pone.0258463.s001.zip › oil rock cores/17.bmp]

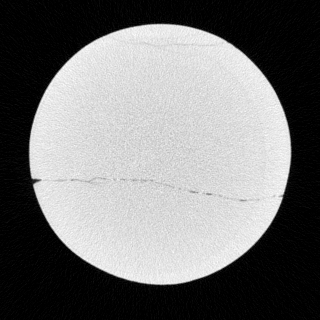

Supplement: S1 File — (ZIP) [file pone.0258463.s001.zip › oil rock cores/18.bmp]

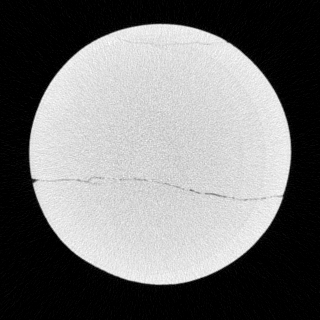

Supplement: S1 File — (ZIP) [file pone.0258463.s001.zip › oil rock cores/19.bmp]

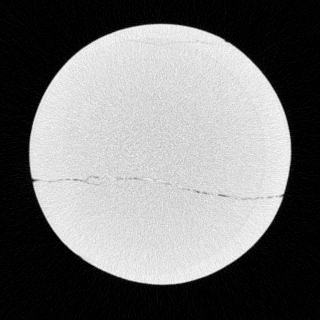

Supplement: S1 File — (ZIP) [file pone.0258463.s001.zip › oil rock cores/20.bmp]

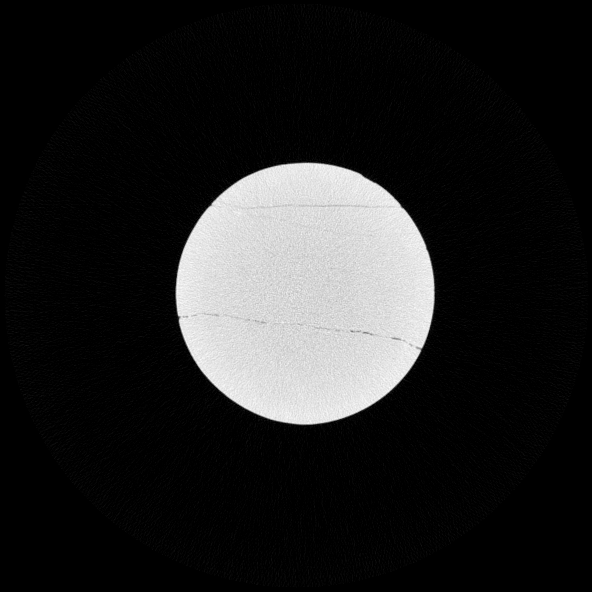

Supplement: S1 File — (ZIP) [file pone.0258463.s001.zip › oil rock cores/201108290013.bmp]

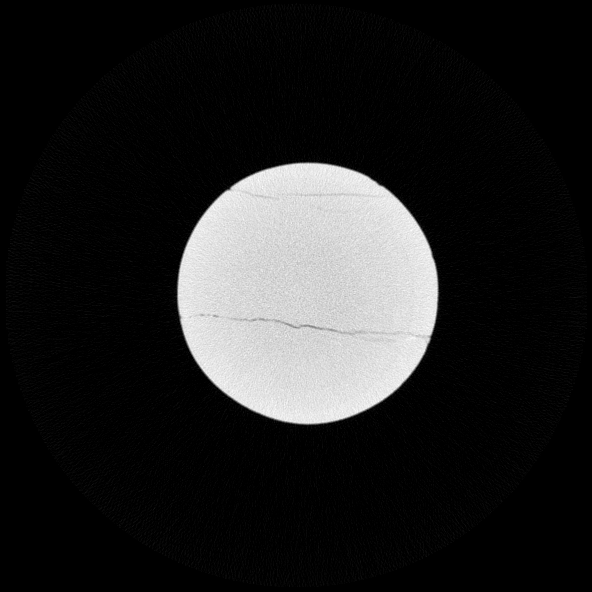

Supplement: S1 File — (ZIP) [file pone.0258463.s001.zip › oil rock cores/201108290014.bmp]

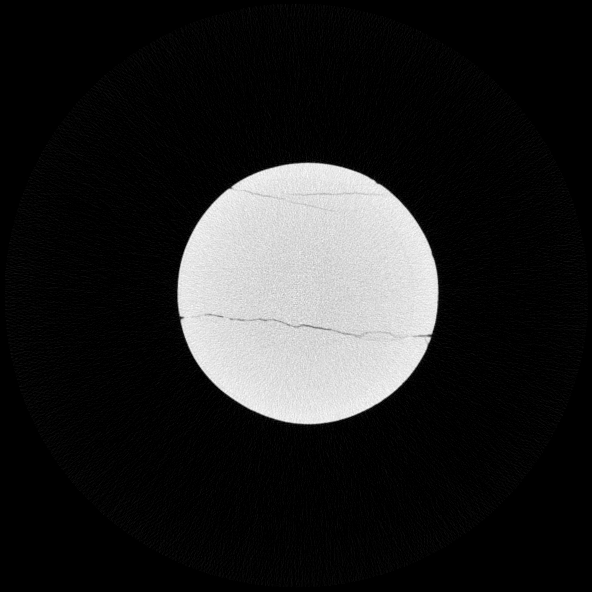

Supplement: S1 File — (ZIP) [file pone.0258463.s001.zip › oil rock cores/201108290015.bmp]

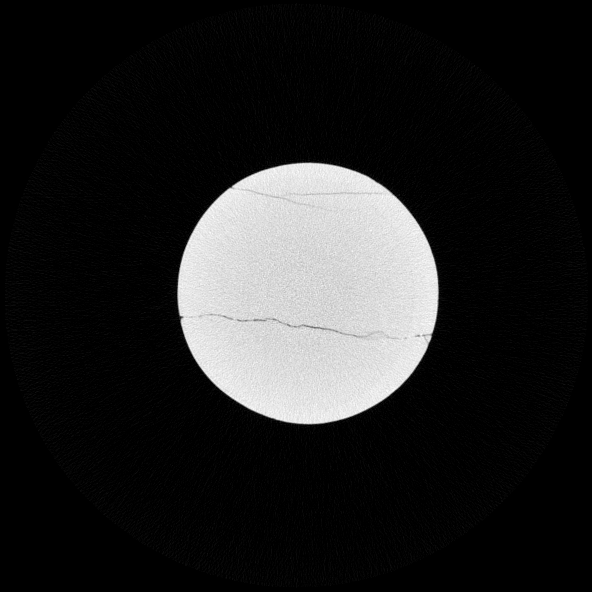

Supplement: S1 File — (ZIP) [file pone.0258463.s001.zip › oil rock cores/201108290016.bmp]

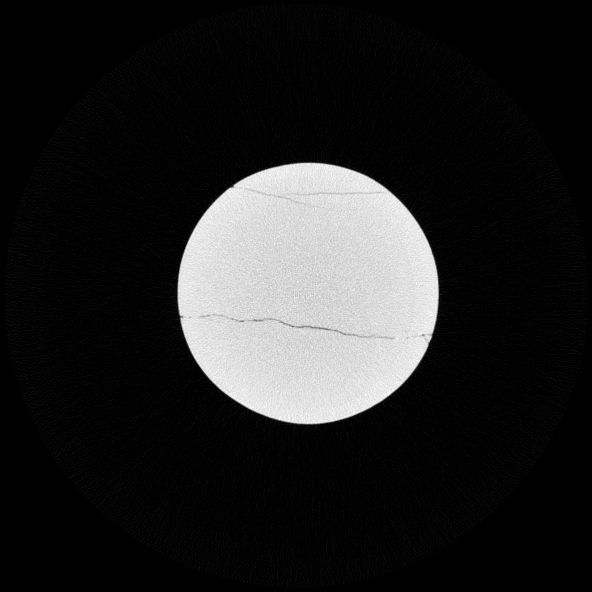

Supplement: S1 File — (ZIP) [file pone.0258463.s001.zip › oil rock cores/201108290017.bmp]

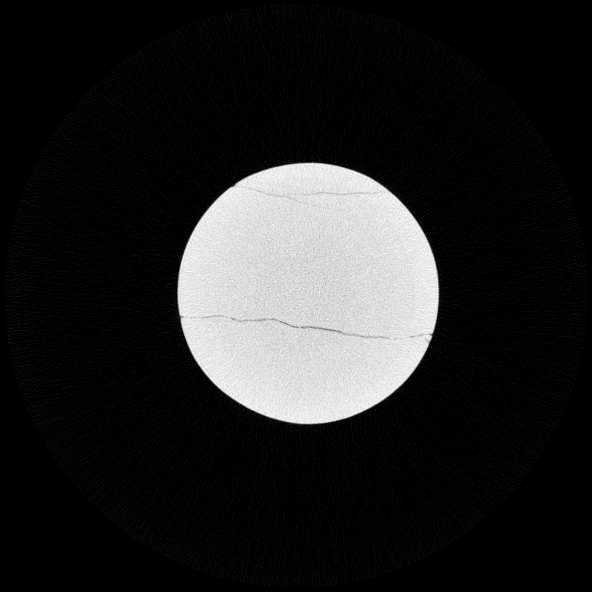

Supplement: S1 File — (ZIP) [file pone.0258463.s001.zip › oil rock cores/201108290018.bmp]

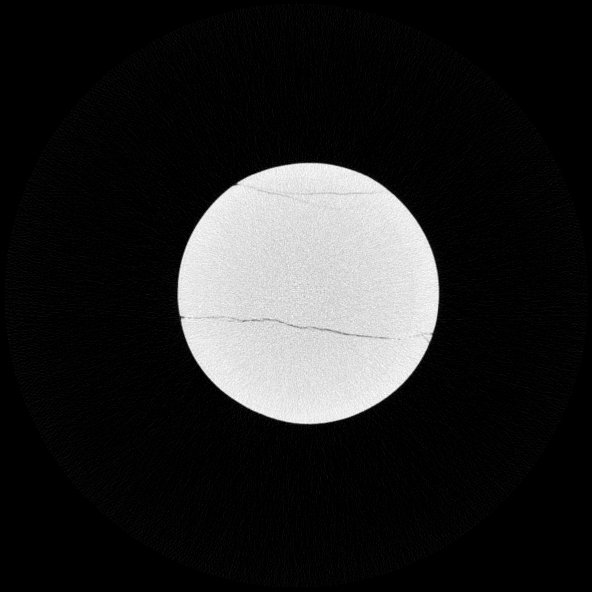

Supplement: S1 File — (ZIP) [file pone.0258463.s001.zip › oil rock cores/201108290019.bmp]

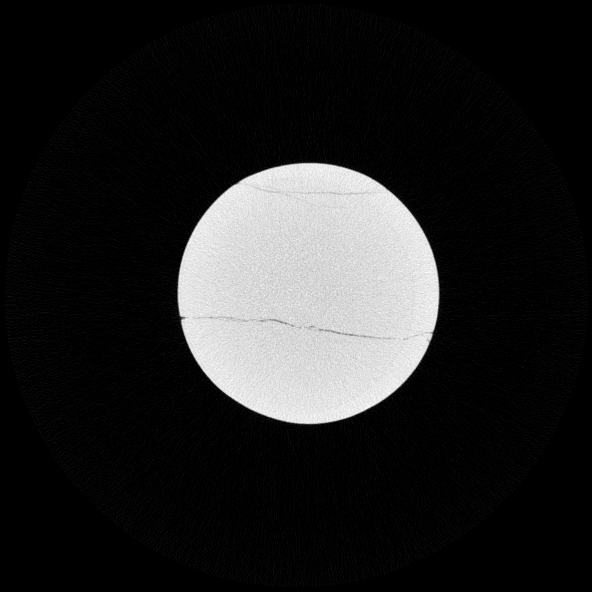

Supplement: S1 File — (ZIP) [file pone.0258463.s001.zip › oil rock cores/201108290020.bmp]

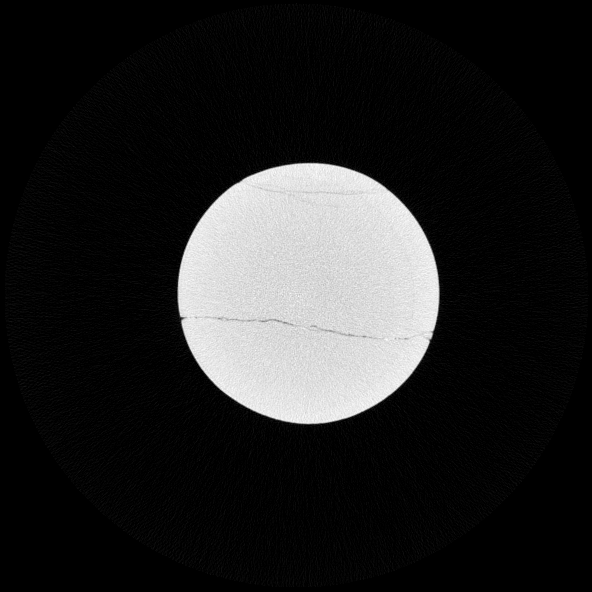

Supplement: S1 File — (ZIP) [file pone.0258463.s001.zip › oil rock cores/201108290021.bmp]

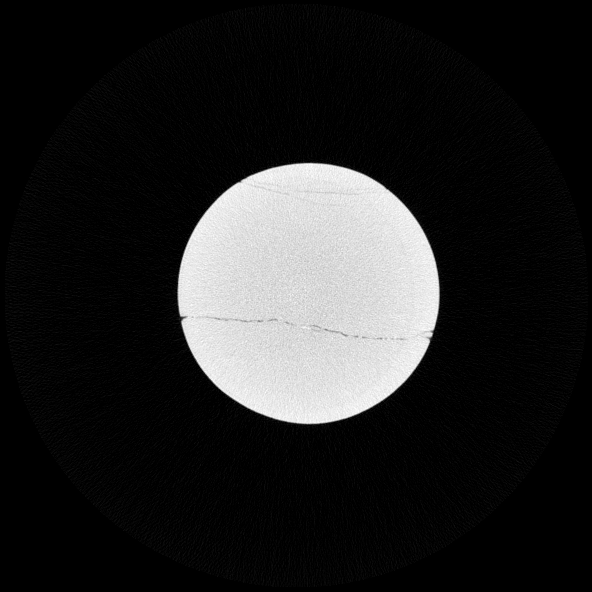

Supplement: S1 File — (ZIP) [file pone.0258463.s001.zip › oil rock cores/201108290022.bmp]

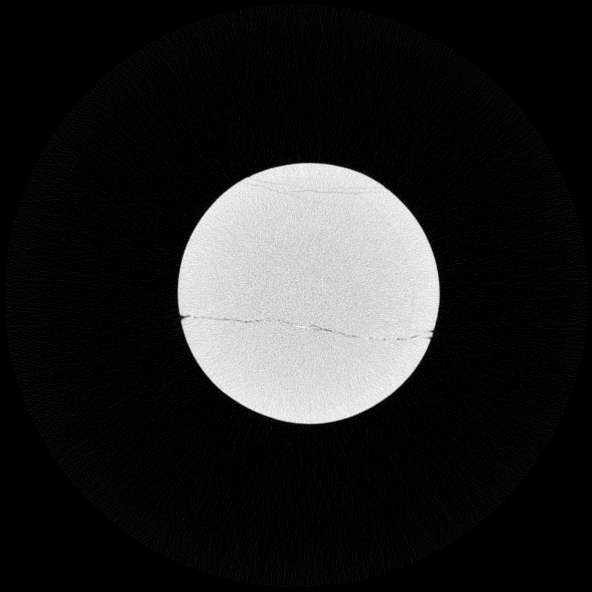

Supplement: S1 File — (ZIP) [file pone.0258463.s001.zip › oil rock cores/201108290023.bmp]

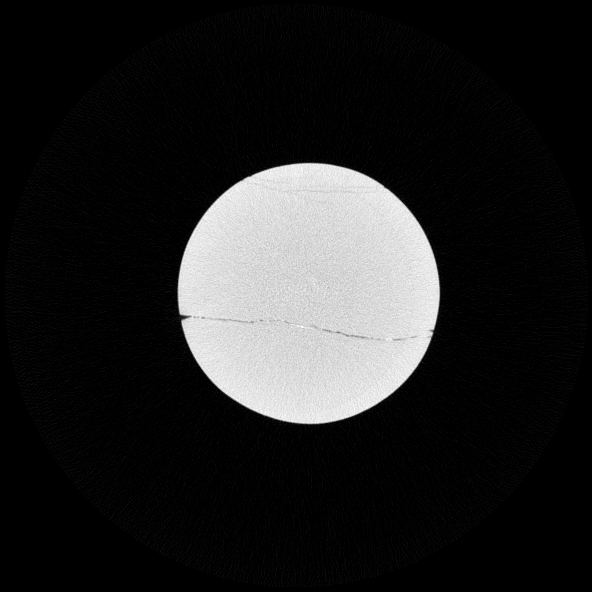

Supplement: S1 File — (ZIP) [file pone.0258463.s001.zip › oil rock cores/201108290024.bmp]

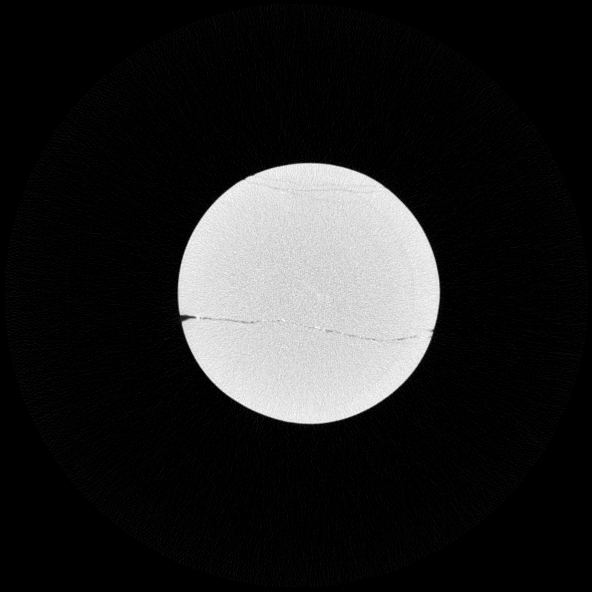

Supplement: S1 File — (ZIP) [file pone.0258463.s001.zip › oil rock cores/201108290025.bmp]

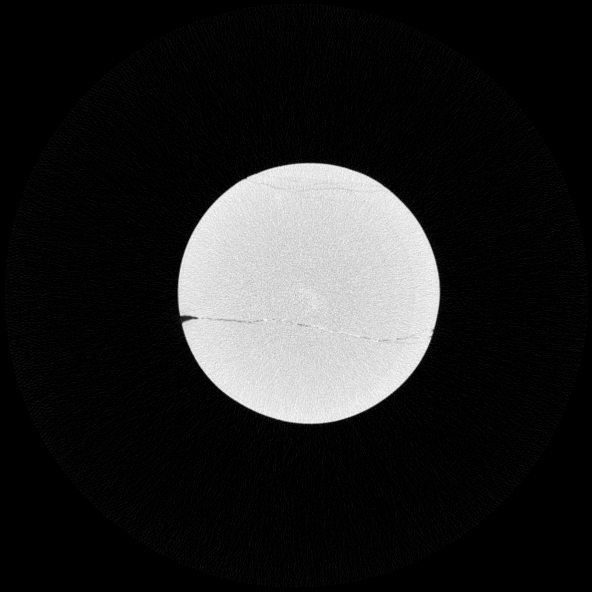

Supplement: S1 File — (ZIP) [file pone.0258463.s001.zip › oil rock cores/201108290026.bmp]

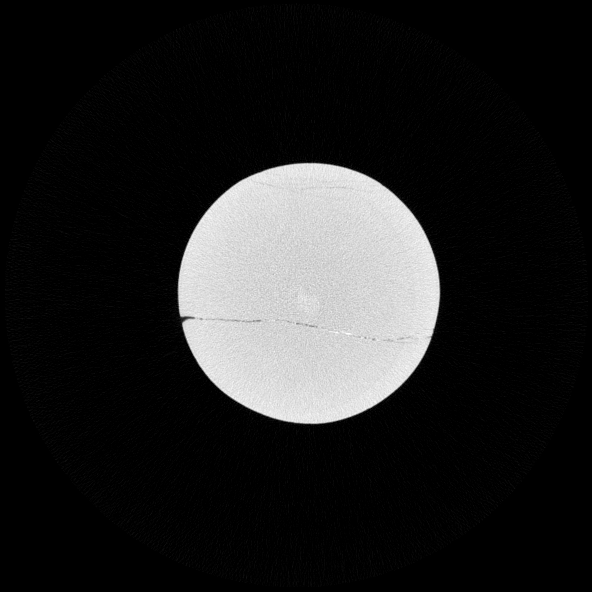

Supplement: S1 File — (ZIP) [file pone.0258463.s001.zip › oil rock cores/201108290027.bmp]

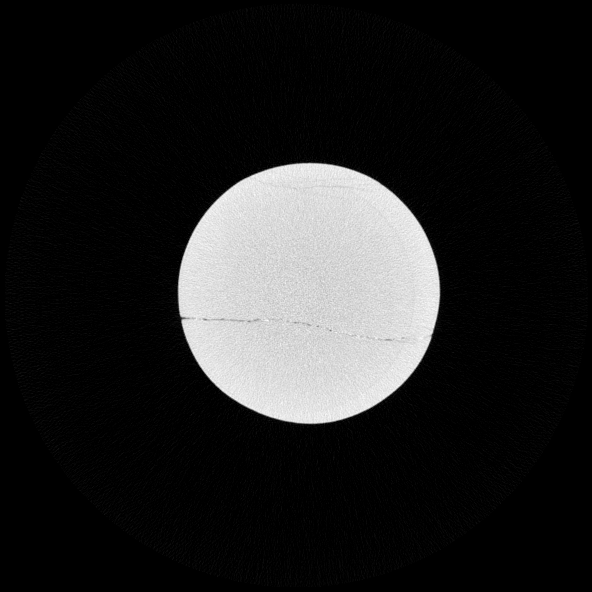

Supplement: S1 File — (ZIP) [file pone.0258463.s001.zip › oil rock cores/201108290028.bmp]

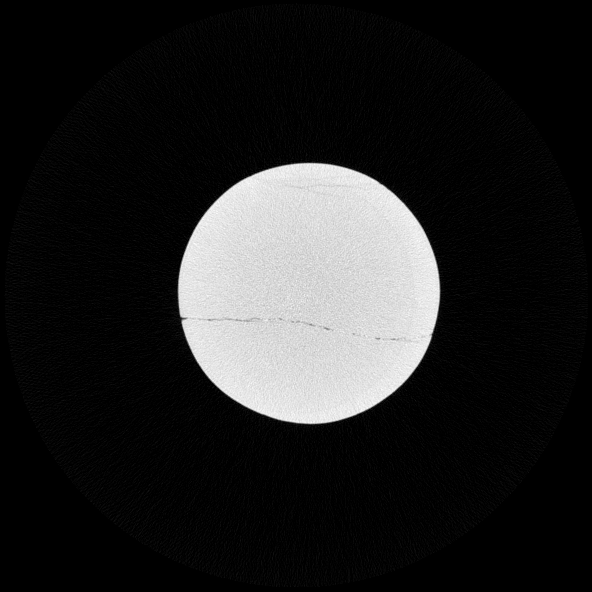

Supplement: S1 File — (ZIP) [file pone.0258463.s001.zip › oil rock cores/201108290029.bmp]

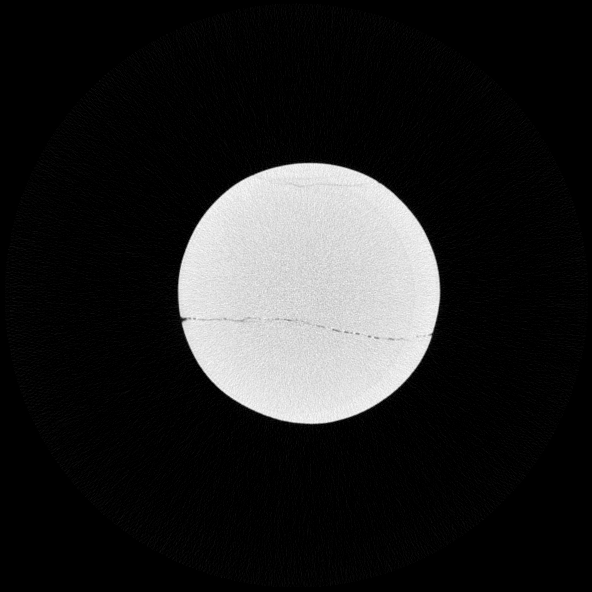

Supplement: S1 File — (ZIP) [file pone.0258463.s001.zip › oil rock cores/201108290030.bmp]

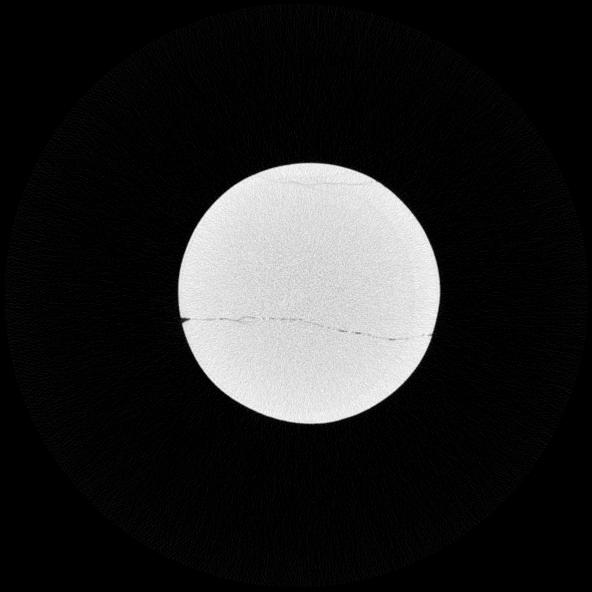

Supplement: S1 File — (ZIP) [file pone.0258463.s001.zip › oil rock cores/201108290031.bmp]

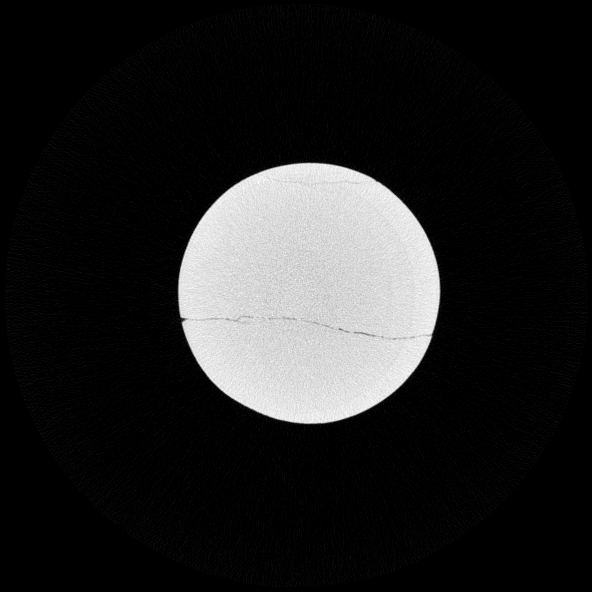

Supplement: S1 File — (ZIP) [file pone.0258463.s001.zip › oil rock cores/201108290032.bmp]

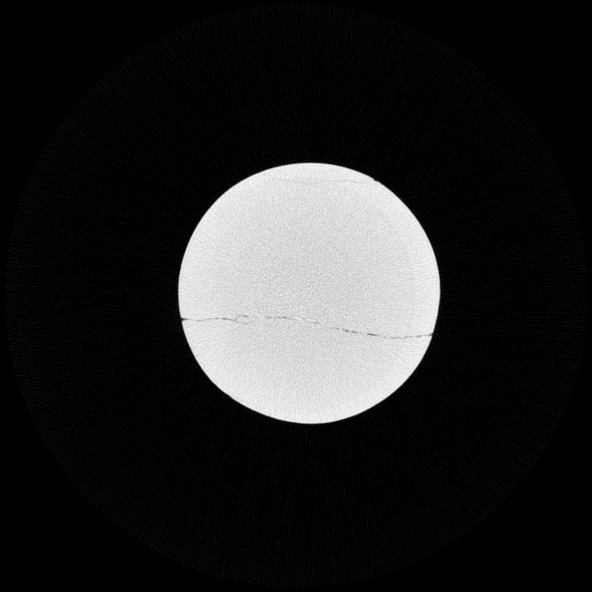

Supplement: S1 File — (ZIP) [file pone.0258463.s001.zip › oil rock cores/201108290033.bmp]

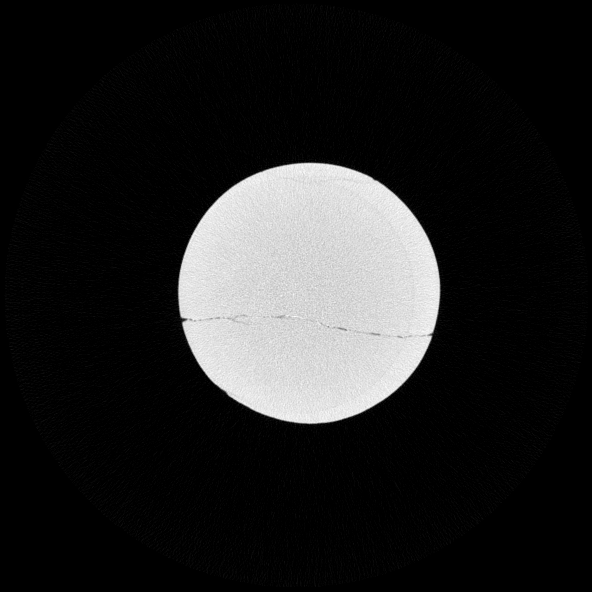

Supplement: S1 File — (ZIP) [file pone.0258463.s001.zip › oil rock cores/201108290034.bmp]

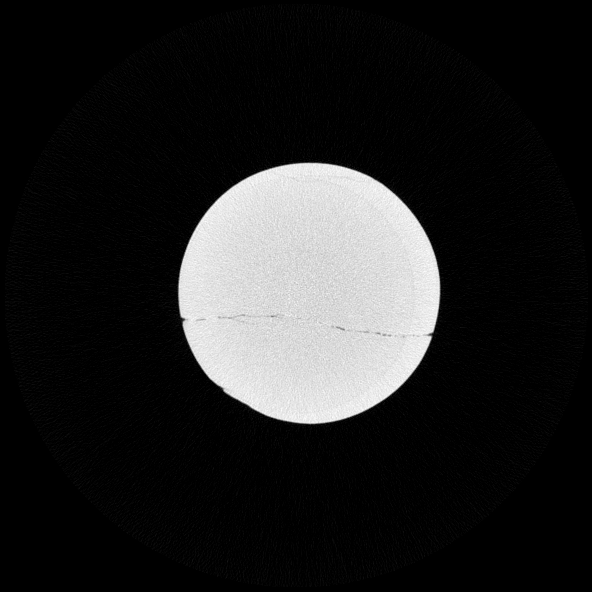

Supplement: S1 File — (ZIP) [file pone.0258463.s001.zip › oil rock cores/201108290035.bmp]

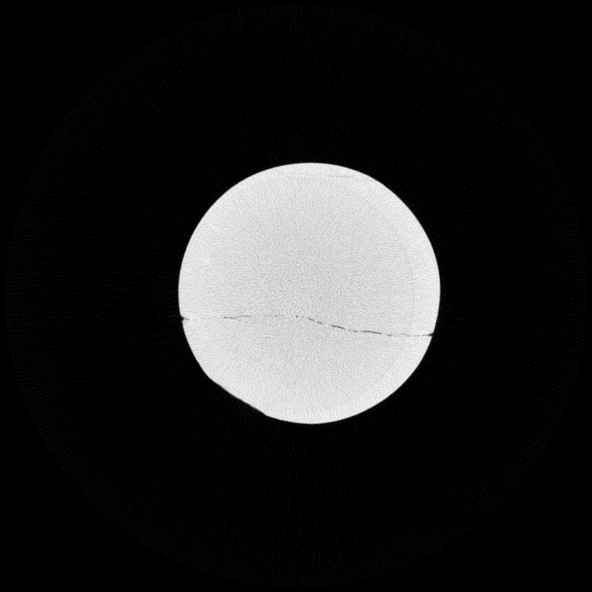

Supplement: S1 File — (ZIP) [file pone.0258463.s001.zip › oil rock cores/201108290036.bmp]

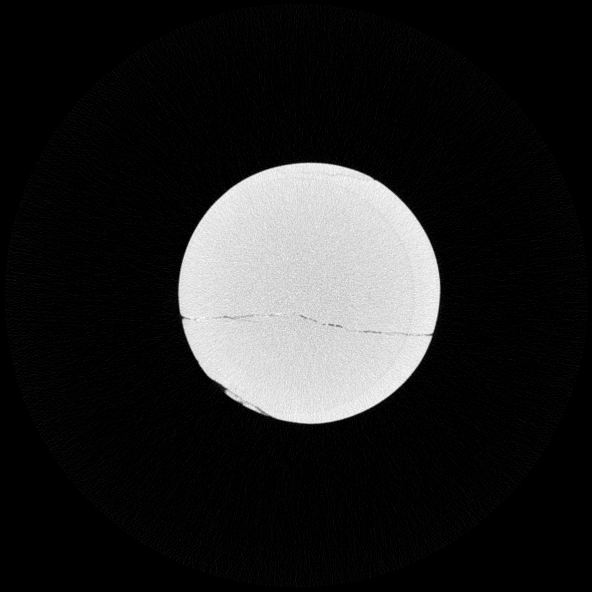

Supplement: S1 File — (ZIP) [file pone.0258463.s001.zip › oil rock cores/201108290037.bmp]

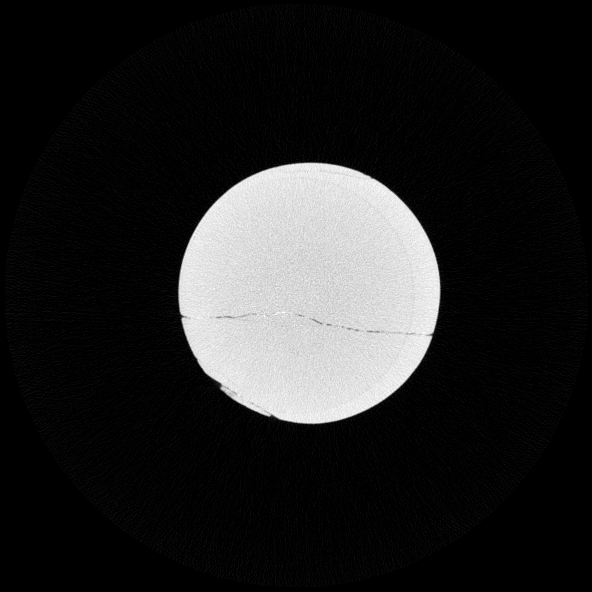

Supplement: S1 File — (ZIP) [file pone.0258463.s001.zip › oil rock cores/201108290038.bmp]

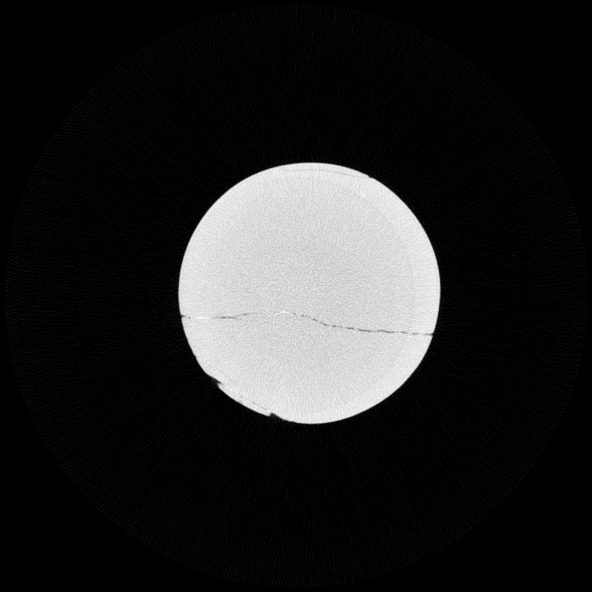

Supplement: S1 File — (ZIP) [file pone.0258463.s001.zip › oil rock cores/201108290039.bmp]

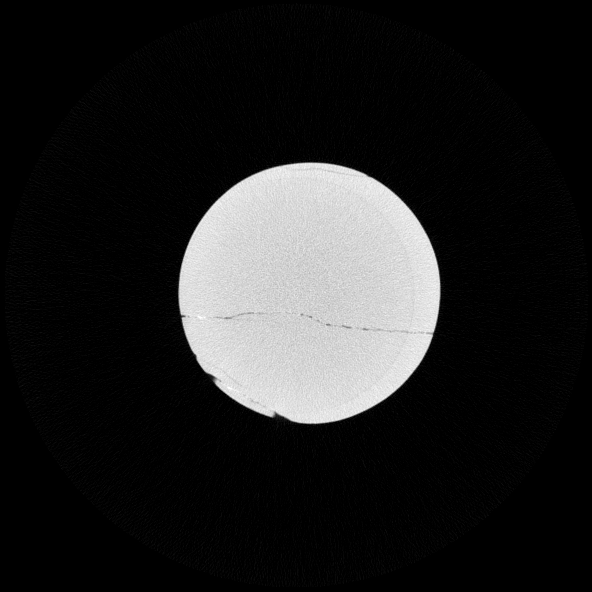

Supplement: S1 File — (ZIP) [file pone.0258463.s001.zip › oil rock cores/201108290040.bmp]

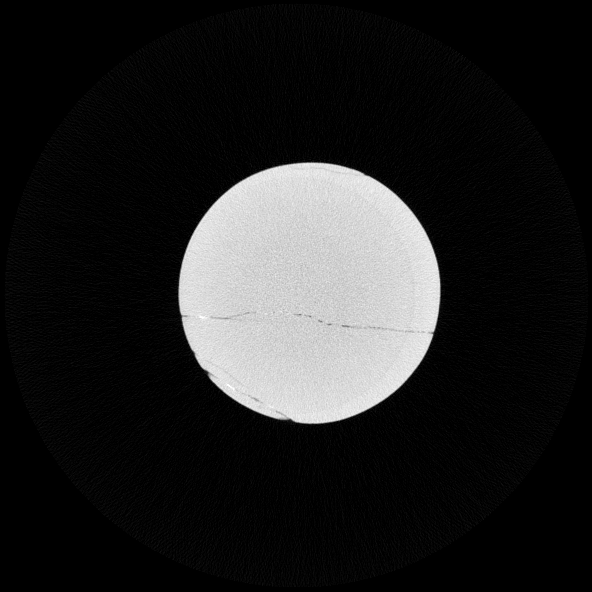

Supplement: S1 File — (ZIP) [file pone.0258463.s001.zip › oil rock cores/201108290041.bmp]

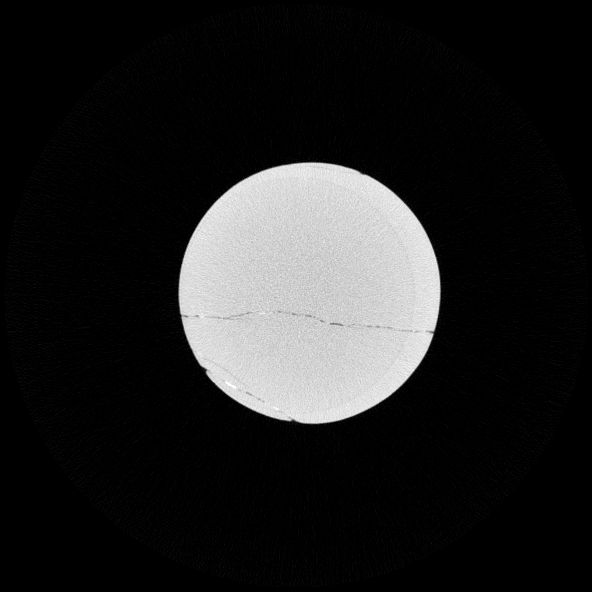

Supplement: S1 File — (ZIP) [file pone.0258463.s001.zip › oil rock cores/201108290042.bmp]

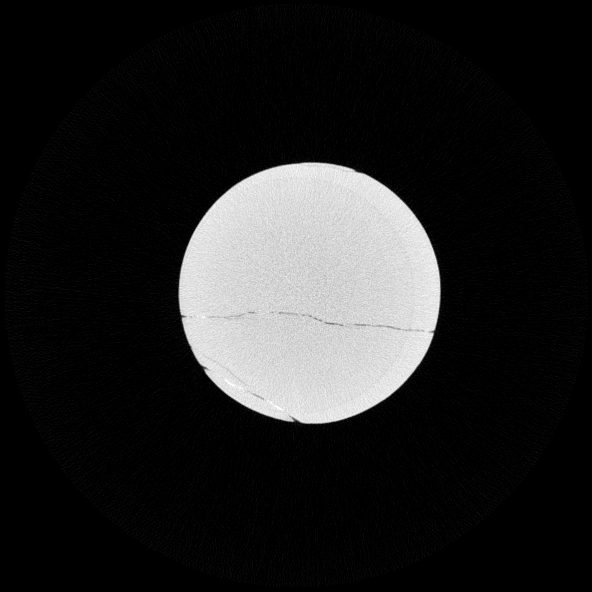

Supplement: S1 File — (ZIP) [file pone.0258463.s001.zip › oil rock cores/201108290043.bmp]

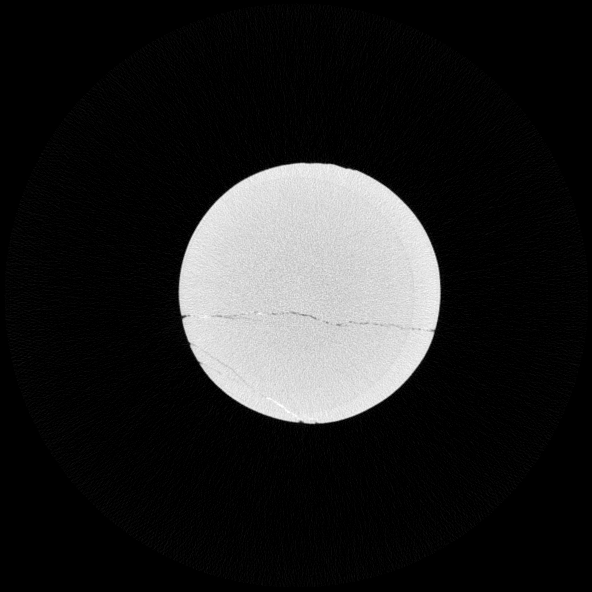

Supplement: S1 File — (ZIP) [file pone.0258463.s001.zip › oil rock cores/201108290044.bmp]

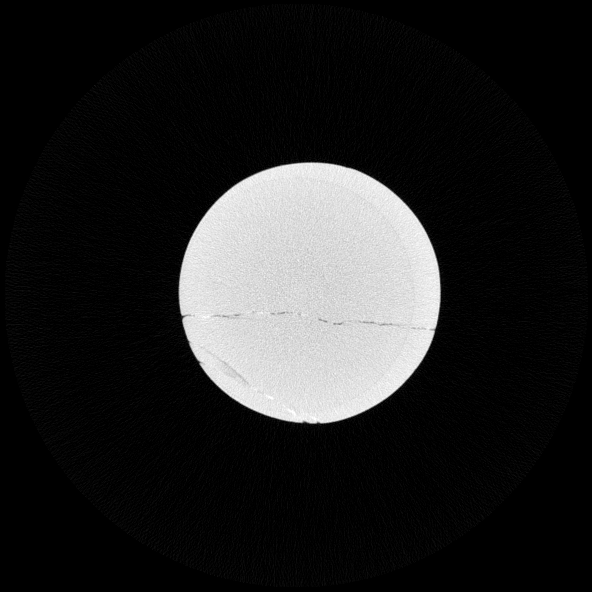

Supplement: S1 File — (ZIP) [file pone.0258463.s001.zip › oil rock cores/201108290045.bmp]

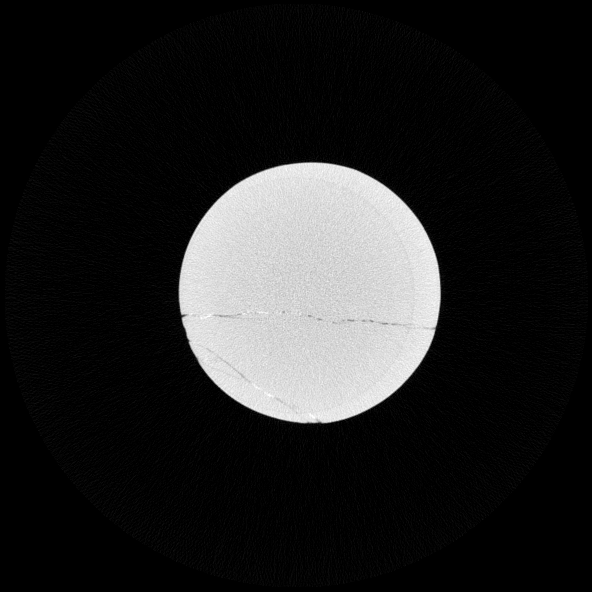

Supplement: S1 File — (ZIP) [file pone.0258463.s001.zip › oil rock cores/201108290046.bmp]

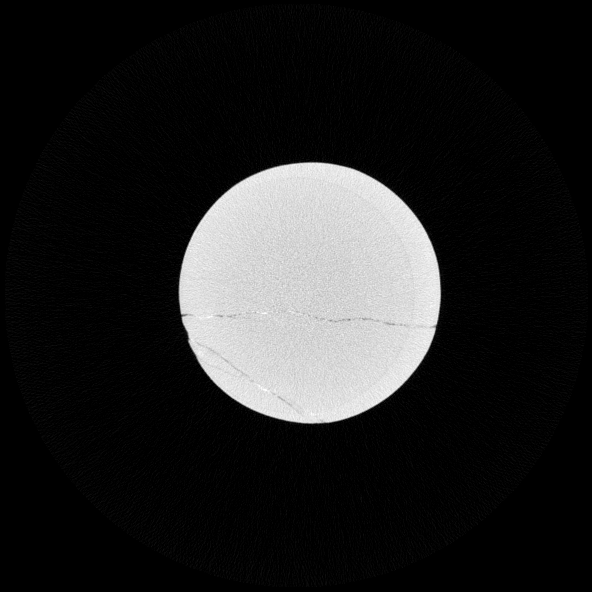

Supplement: S1 File — (ZIP) [file pone.0258463.s001.zip › oil rock cores/201108290047.bmp]

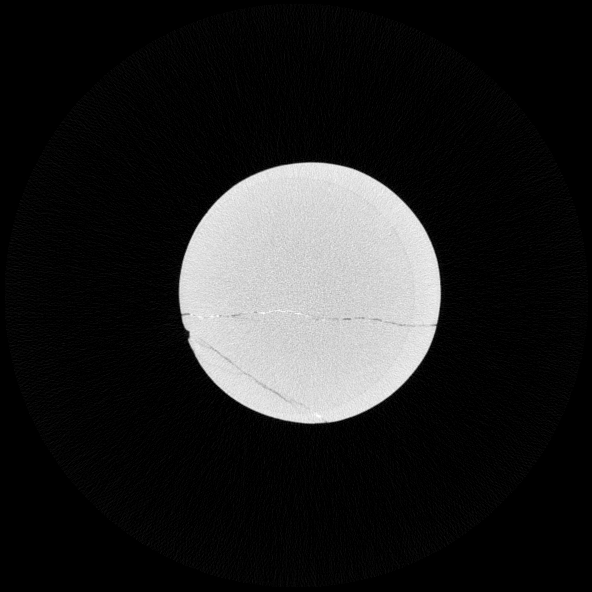

Supplement: S1 File — (ZIP) [file pone.0258463.s001.zip › oil rock cores/201108290048.bmp]

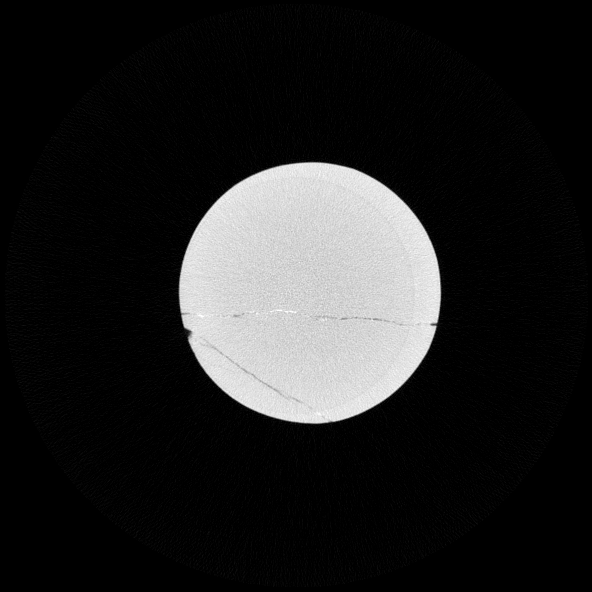

Supplement: S1 File — (ZIP) [file pone.0258463.s001.zip › oil rock cores/201108290049.bmp]

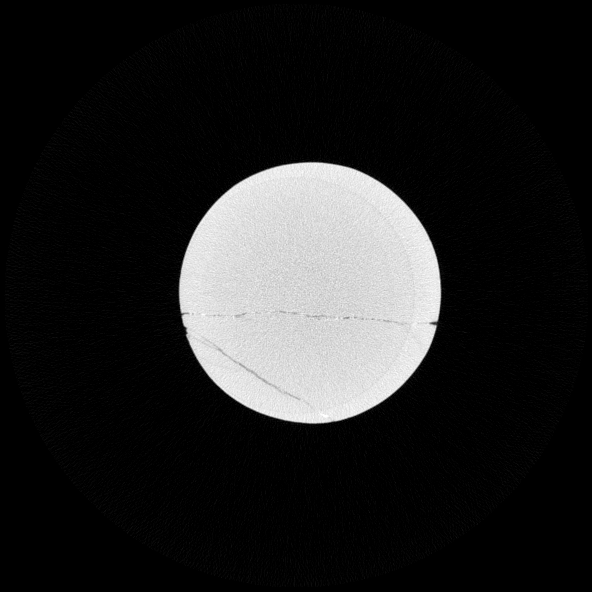

Supplement: S1 File — (ZIP) [file pone.0258463.s001.zip › oil rock cores/201108290050.bmp]

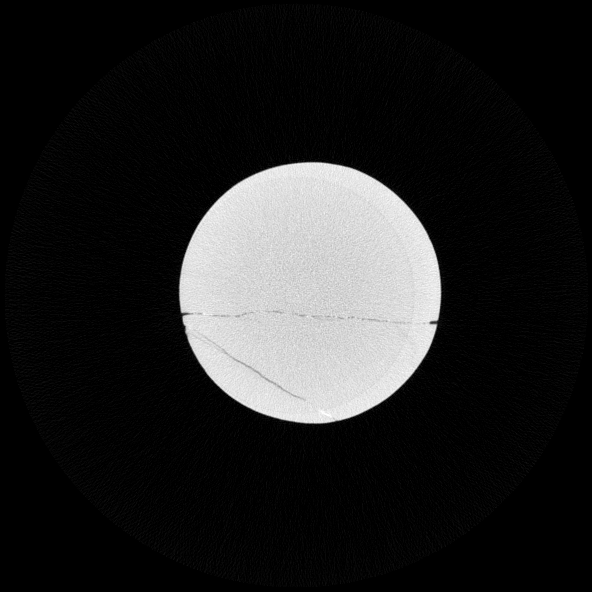

Supplement: S1 File — (ZIP) [file pone.0258463.s001.zip › oil rock cores/201108290051.bmp]

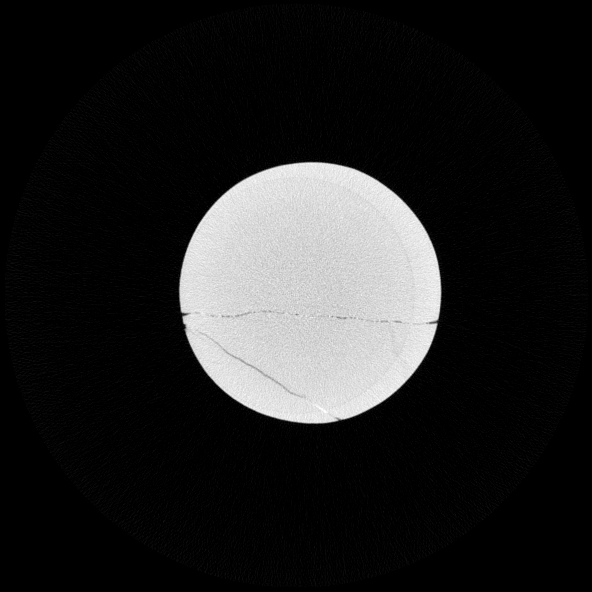

Supplement: S1 File — (ZIP) [file pone.0258463.s001.zip › oil rock cores/201108290052.bmp]

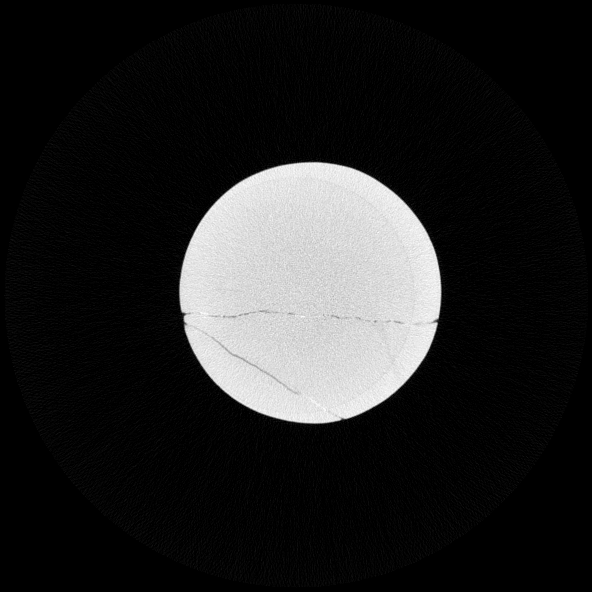

Supplement: S1 File — (ZIP) [file pone.0258463.s001.zip › oil rock cores/201108290053.bmp]

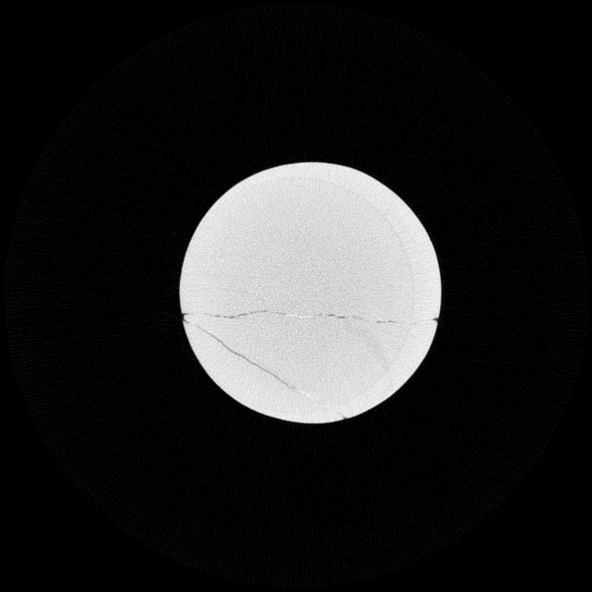

Supplement: S1 File — (ZIP) [file pone.0258463.s001.zip › oil rock cores/201108290054.bmp]

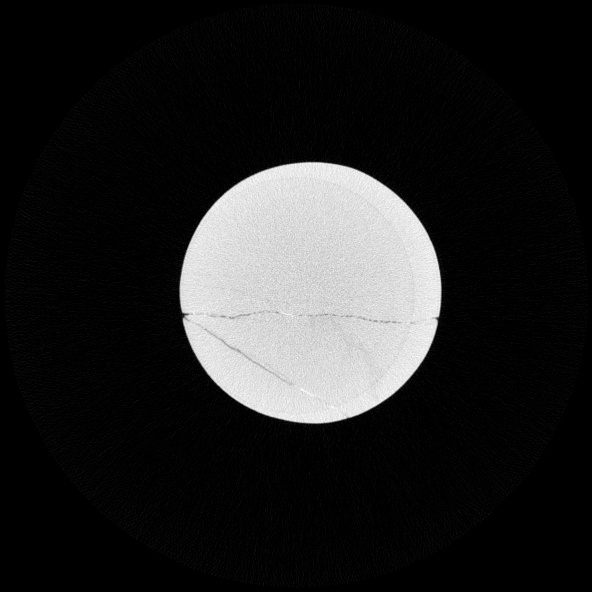

Supplement: S1 File — (ZIP) [file pone.0258463.s001.zip › oil rock cores/201108290055.bmp]

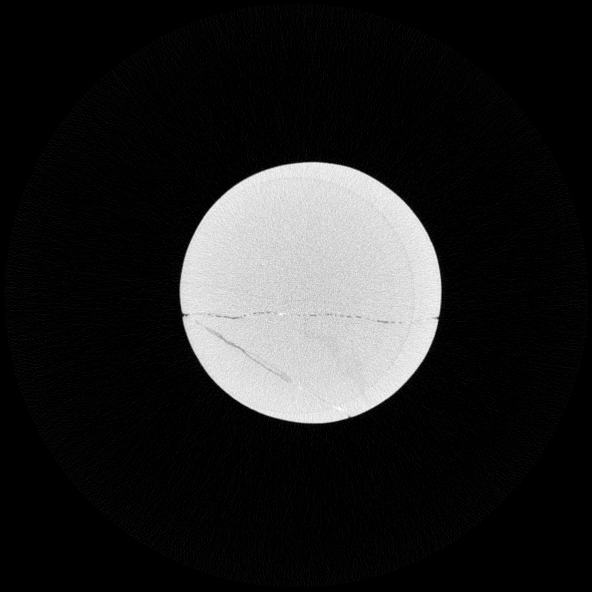

Supplement: S1 File — (ZIP) [file pone.0258463.s001.zip › oil rock cores/201108290056.bmp]

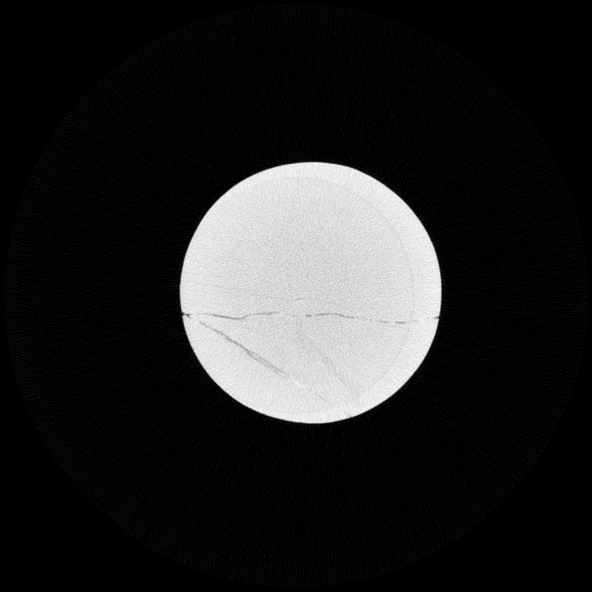

Supplement: S1 File — (ZIP) [file pone.0258463.s001.zip › oil rock cores/201108290057.bmp]

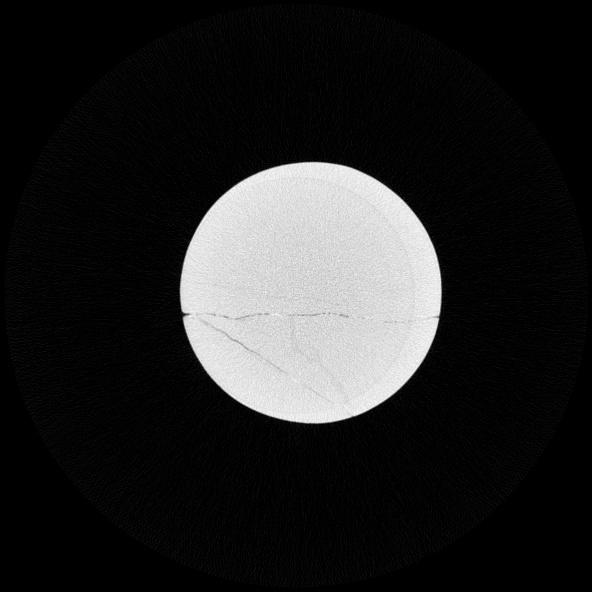

Supplement: S1 File — (ZIP) [file pone.0258463.s001.zip › oil rock cores/201108290058.bmp]

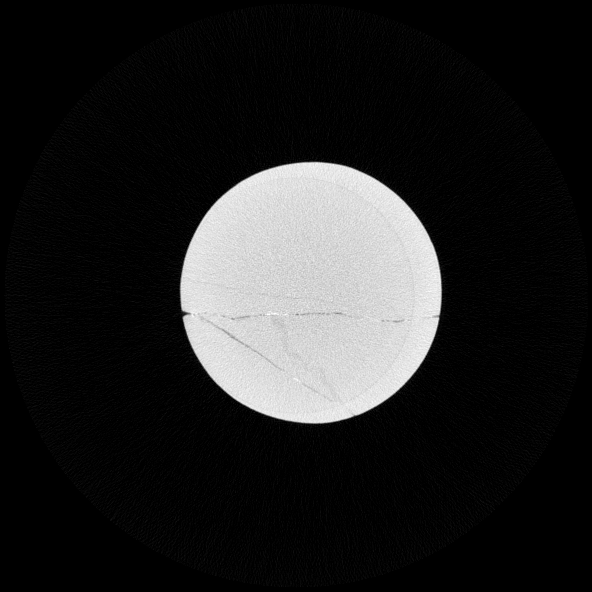

Supplement: S1 File — (ZIP) [file pone.0258463.s001.zip › oil rock cores/201108290059.bmp]

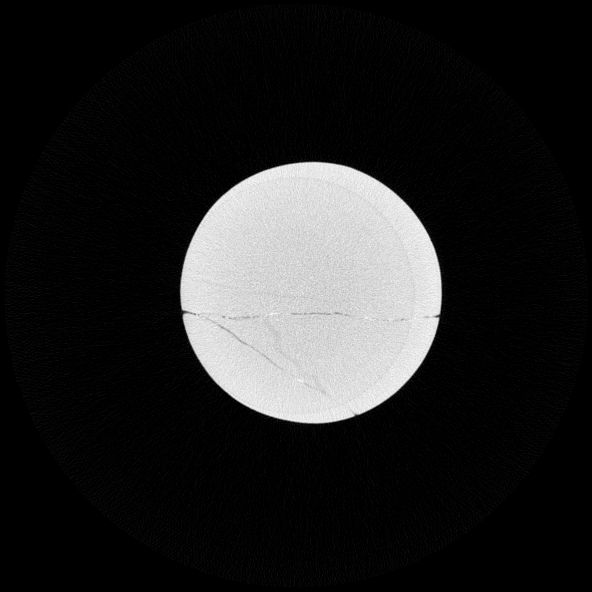

Supplement: S1 File — (ZIP) [file pone.0258463.s001.zip › oil rock cores/201108290060.bmp]

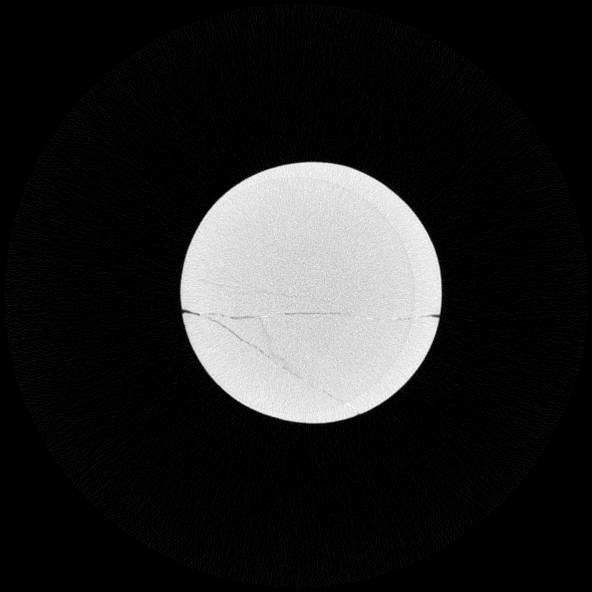

Supplement: S1 File — (ZIP) [file pone.0258463.s001.zip › oil rock cores/201108290061.bmp]

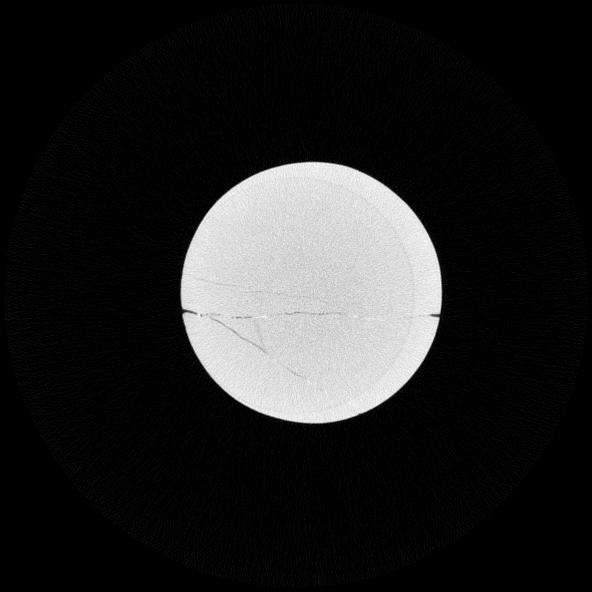

Supplement: S1 File — (ZIP) [file pone.0258463.s001.zip › oil rock cores/201108290062.bmp]

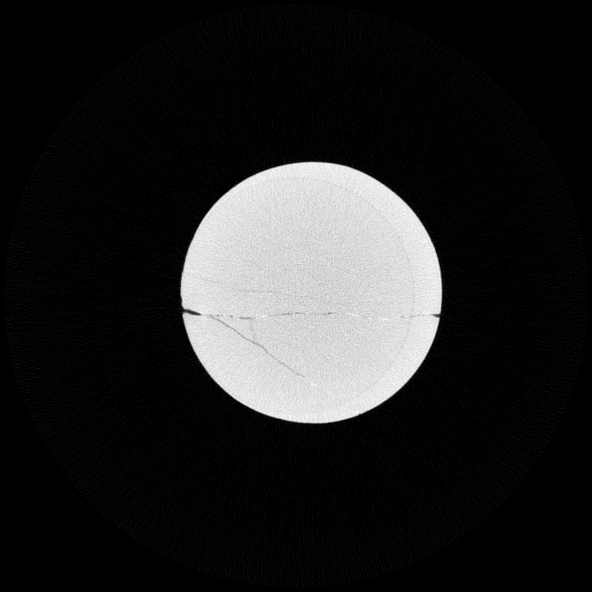

Supplement: S1 File — (ZIP) [file pone.0258463.s001.zip › oil rock cores/201108290063.bmp]

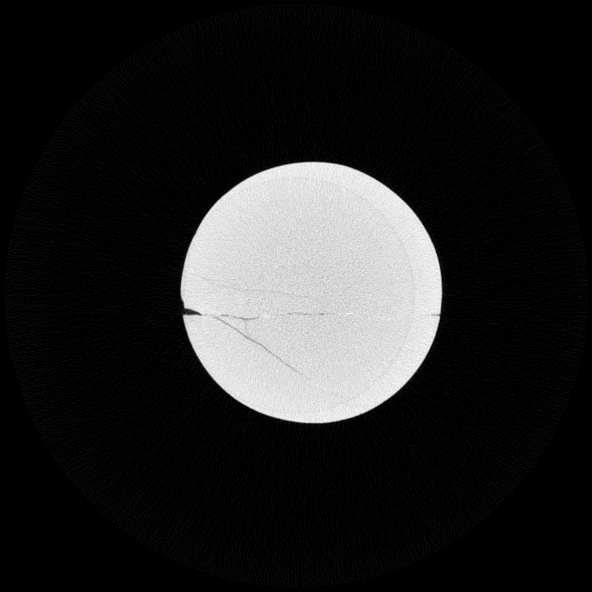

Supplement: S1 File — (ZIP) [file pone.0258463.s001.zip › oil rock cores/201108290064.bmp]

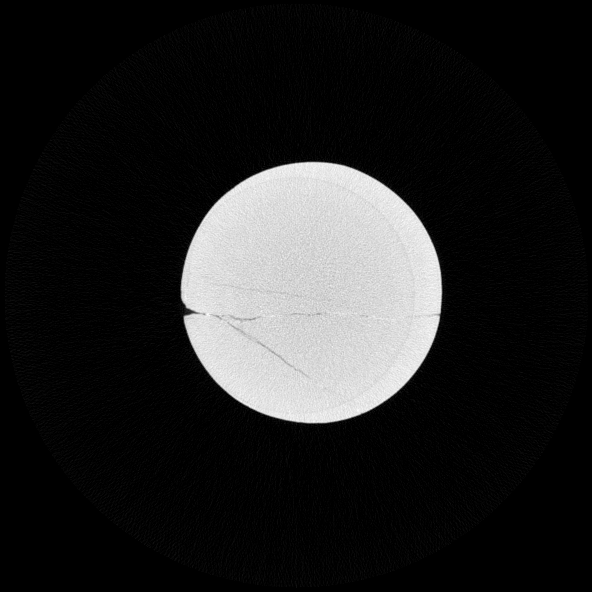

Supplement: S1 File — (ZIP) [file pone.0258463.s001.zip › oil rock cores/201108290065.bmp]

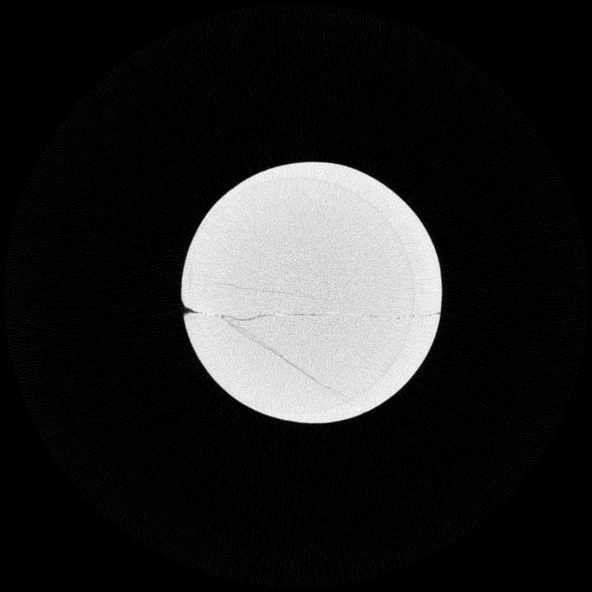

Supplement: S1 File — (ZIP) [file pone.0258463.s001.zip › oil rock cores/201108290066.bmp]

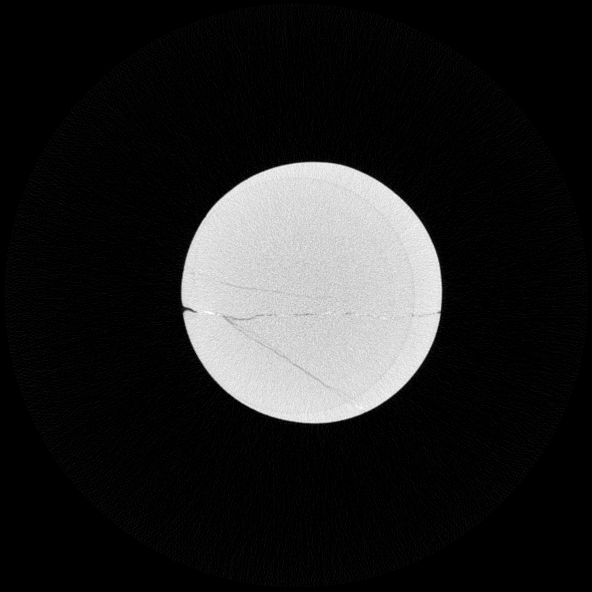

Supplement: S1 File — (ZIP) [file pone.0258463.s001.zip › oil rock cores/201108290067.bmp]

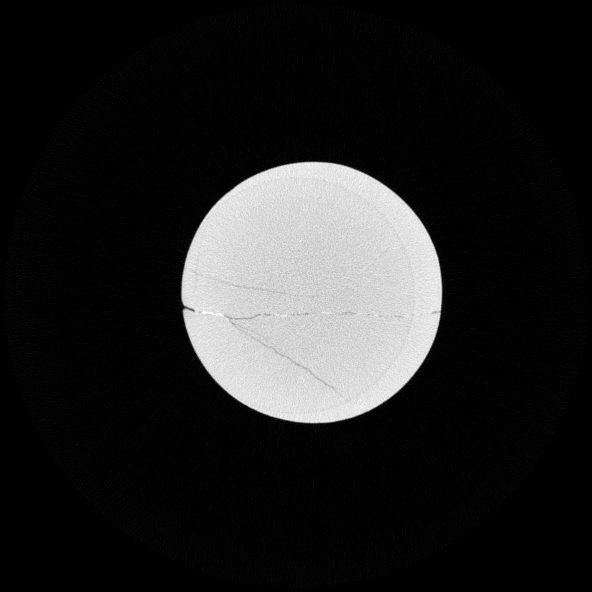

Supplement: S1 File — (ZIP) [file pone.0258463.s001.zip › oil rock cores/201108290068.bmp]

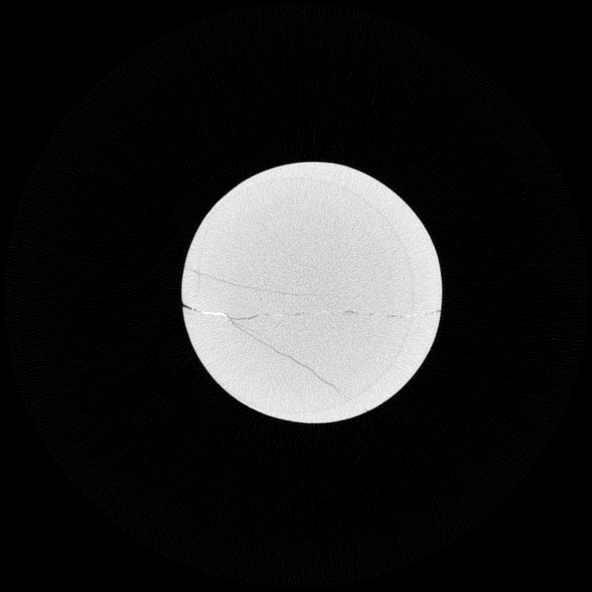

Supplement: S1 File — (ZIP) [file pone.0258463.s001.zip › oil rock cores/201108290069.bmp]

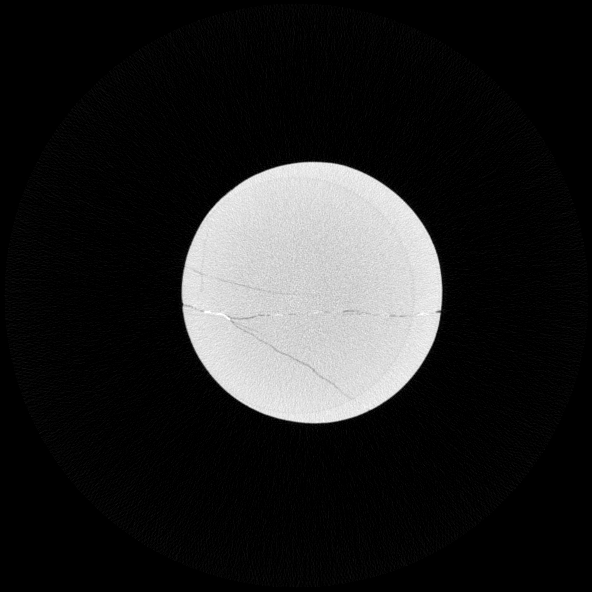

Supplement: S1 File — (ZIP) [file pone.0258463.s001.zip › oil rock cores/201108290070.bmp]

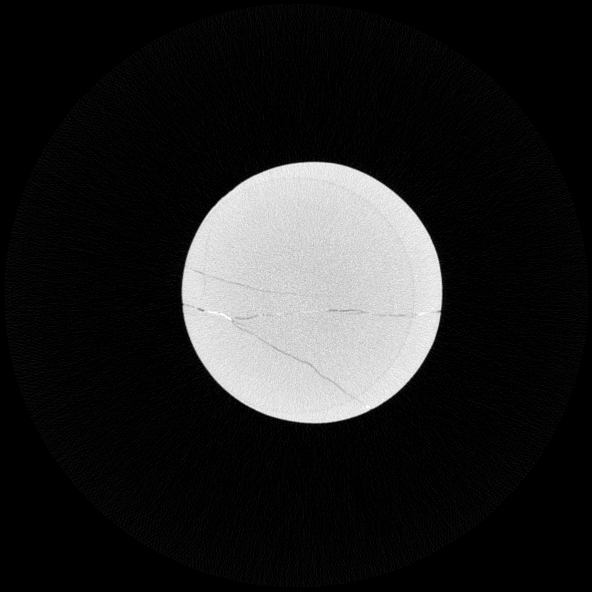

Supplement: S1 File — (ZIP) [file pone.0258463.s001.zip › oil rock cores/201108290071.bmp]

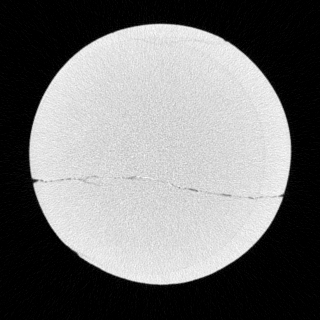

Supplement: S1 File — (ZIP) [file pone.0258463.s001.zip › oil rock cores/21.bmp]

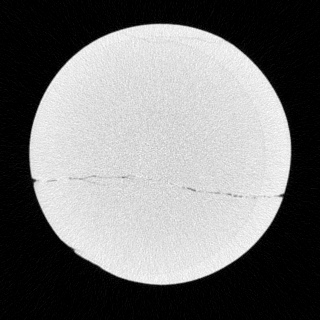

Supplement: S1 File — (ZIP) [file pone.0258463.s001.zip › oil rock cores/22.bmp]

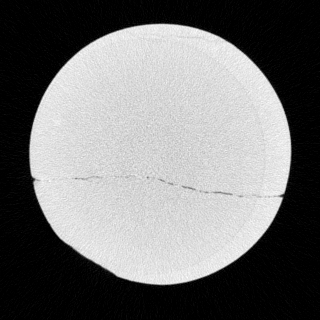

Supplement: S1 File — (ZIP) [file pone.0258463.s001.zip › oil rock cores/23.bmp]

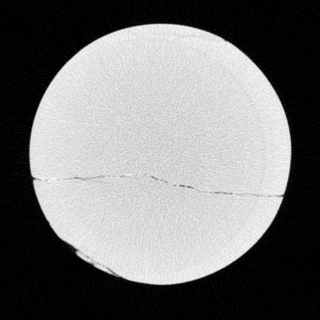

Supplement: S1 File — (ZIP) [file pone.0258463.s001.zip › oil rock cores/24.bmp]

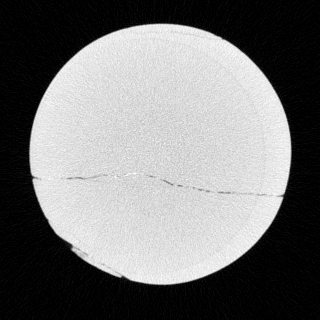

Supplement: S1 File — (ZIP) [file pone.0258463.s001.zip › oil rock cores/25.bmp]

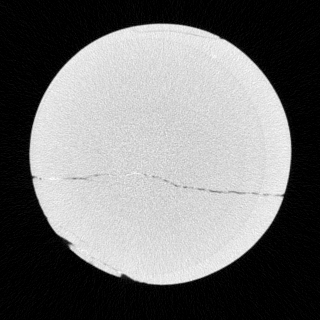

Supplement: S1 File — (ZIP) [file pone.0258463.s001.zip › oil rock cores/26.bmp]

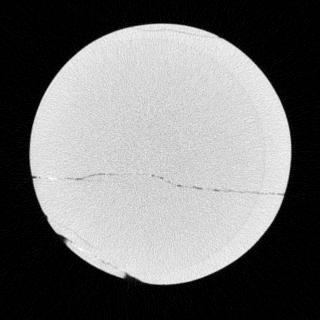

Supplement: S1 File — (ZIP) [file pone.0258463.s001.zip › oil rock cores/27.bmp]

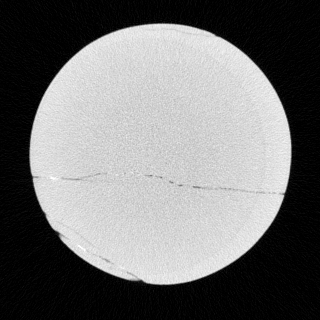

Supplement: S1 File — (ZIP) [file pone.0258463.s001.zip › oil rock cores/28.bmp]

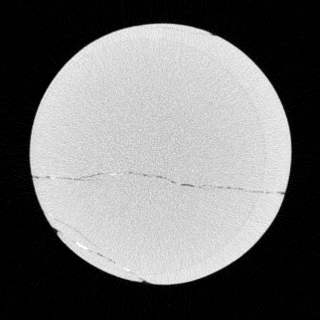

Supplement: S1 File — (ZIP) [file pone.0258463.s001.zip › oil rock cores/29.bmp]

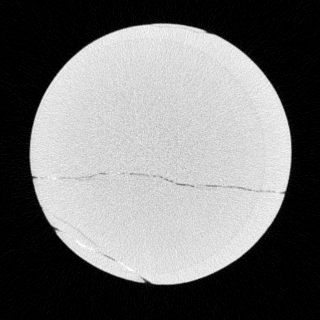

Supplement: S1 File — (ZIP) [file pone.0258463.s001.zip › oil rock cores/30.bmp]

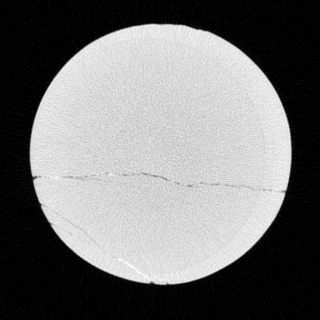

Supplement: S1 File — (ZIP) [file pone.0258463.s001.zip › oil rock cores/31.bmp]

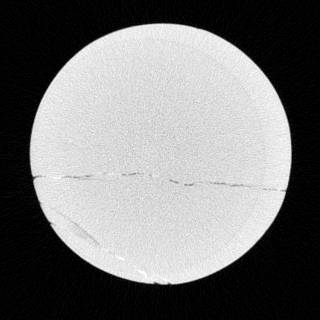

Supplement: S1 File — (ZIP) [file pone.0258463.s001.zip › oil rock cores/32.bmp]

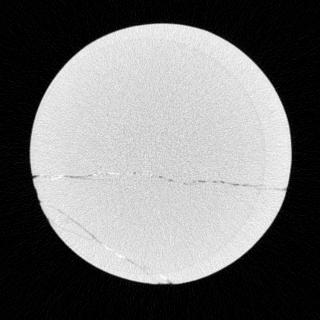

Supplement: S1 File — (ZIP) [file pone.0258463.s001.zip › oil rock cores/33.bmp]

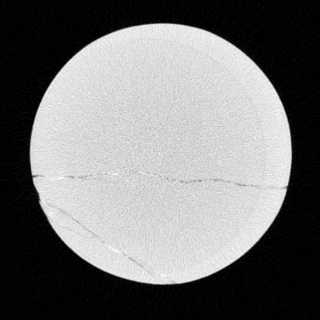

Supplement: S1 File — (ZIP) [file pone.0258463.s001.zip › oil rock cores/34.bmp]

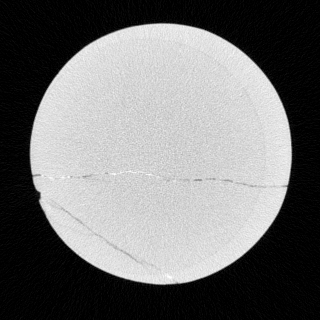

Supplement: S1 File — (ZIP) [file pone.0258463.s001.zip › oil rock cores/35.bmp]

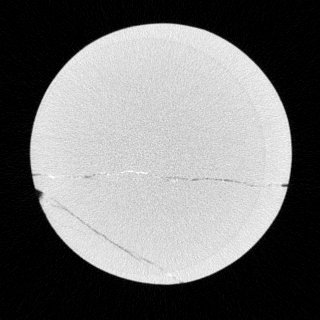

Supplement: S1 File — (ZIP) [file pone.0258463.s001.zip › oil rock cores/36.bmp]

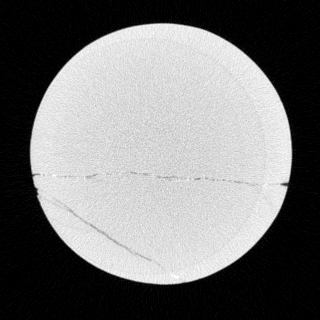

Supplement: S1 File — (ZIP) [file pone.0258463.s001.zip › oil rock cores/37.bmp]

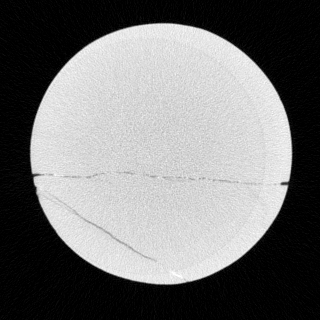

Supplement: S1 File — (ZIP) [file pone.0258463.s001.zip › oil rock cores/38.bmp]

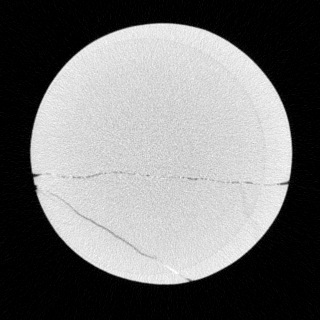

Supplement: S1 File — (ZIP) [file pone.0258463.s001.zip › oil rock cores/39.bmp]

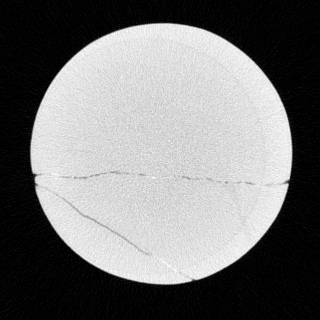

Supplement: S1 File — (ZIP) [file pone.0258463.s001.zip › oil rock cores/40.bmp]
